# Full Conjugation in a Polymer with Non‐conjugated Piperazine‐2,5‐dione Units via Energy‐minimized Lactam‐to‐Lactim Tautomerization Enables Water‐gated Transistor Fluoride Sensors

**DOI:** 10.1002/anie.202419314

**Published:** 2024-12-05

**Authors:** Naixin Zhao, Sung Jae Jeon, Yi Yuan, Samala Venkateswarlu, Andrew Stella, Jimmy Papazotos, Yuning Li

**Affiliations:** ^1^ Department of Chemical Engineering Waterloo Institute for Nanotechnology (WIN) University of Waterloo 200 University Ave West Waterloo Ontario N2L 3G1 Canada

**Keywords:** lactam-lactim tautomerization, π-conjugated polymers, vinylogous effects, water-gated organic field effect transistors, chemical sensing

## Abstract

Piperazine‐2,5‐dione (glycine anhydride, GA) has recently emerged as a valuable precursor for high‐performance π‐conjugated polymer semiconductors in organic electronics. We utilized GA to design a novel bisindolin‐dihydropiperazine (IDHP)‐based conjugated polymer, PIDHPTT, for aqueous chemical sensing. In the isatin‐flanked monomer, GA exists as a non‐conjugated lactam (DHP‐NH) but converts to a conjugated lactim (DHP‐OH) form within the polymer. Density functional theory (DFT) calculations show that this conversion is driven by energy minimization via extended π‐conjugation. Neighboring DHP units in the lactim form facilitate this process through π‐bridges, demonstrating a vinylogous effect, which has previously only been observed in small molecules. This is the first study to report such a long‐range vinylogous effect in a polymer due to the collective synergy of numerous functional groups. The OH groups in the lactim DHP interact more strongly with fluoride ions than other halides. PIDHPTT exhibits significant changes in optical absorption, electrochemical impedance, and charge transport in response to fluoride ions, which differ from responses to other halides. A water‐gated organic field‐effect transistor based on PIDHPTT shows excellent sensitivity and selectivity for fluoride ions, demonstrating the potential of this polymer design for chemical sensing applications.

## Graphical abstract

We discovered that the non‐conjugated piperazine‐2,5‐dione unit fully tautomerizes into the conjugated 3,6‐dihydropyrazine‐2,5‐diol unit via a long‐range vinylogous effect, driven by energy minimization through π‐electron delocalization along the polymer backbone. This fully conjugated backbone enhances charge transport, while lactim units strongly interact with fluoride ions, making this polymer effective in water‐gated field‐effect transistor fluoride sensors.

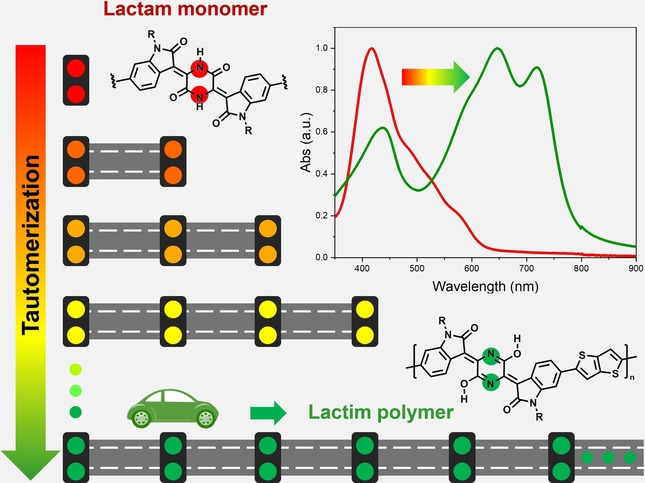

## Introduction

Organic field‐effect transistors (OFETs) are essential components in various organic electronics, including radio frequency identification (RFID) tags, display drivers, and detectors.[^1^, ^2^, ^3^, ^4^, ^5^] Recently, their potential as sensors has gained increasing attention due to their excellent capabilities in detecting various chemical and physical stimuli, remarkable cost‐effectiveness, ability to integrate with substrates of irregular form factors, and mechanical robustness.[^3^, ^5^, ^6^, ^7^, ^8^, ^9^] They are anticipated for a wide range of applications, including the Internet of Things (IoTs), wearable electronics, point‐of‐care diagnostic devices, and intelligent packaging.[^10^, ^11^, ^12^, ^13^, ^14^, ^15^] The sensing mechanisms of OFET‐based chemical sensors rely on complex interactions between the analyte species and the organic semiconductor layer, such as hydrogen‐bonding, charge transfer, and dipole‐dipole interactions.[^16^, ^17^] Developing new functional polymer semiconductors is necessary to clarify these sensing mechanisms and achieve enhanced sensitivity, selectivity, and long‐term stability. Compared to two‐terminal resistor‐based sensors, OFET‐based sensors offer facile signal amplification and tuning by controlling the gate voltage.[^16^, ^18^, ^19^] Additionally, they provide multiple sensory signal parameters, including source‐drain current (*I*_DS_), source‐drain voltage (*V*_DS_), onset voltage (*V*_0_), threshold voltage (*V*_T_), and charge carrier mobility (*μ*).^[20]^ The sensing organic semiconductor layer in an OFET‐based chemical sensor typically needs to be exposed to the sensing environment, which may include air, moisture, or even an aqueous solution. Therefore, robust stability of the organic semiconductor layer in ambient air and/or water is mandatory for reliable sensing and long‐term use. However, only a limited number of organic semiconductors have been reported to function properly under these demanding conditions.[^21^, ^22^, ^23^, ^24^, ^25^, ^26^, ^27^]

Among the numerous π‐conjugated building blocks for polymer semiconductors, structures containing amide/imide auxiliary groups, such as diketopyrrolopyrrole (DPP), isoindigo, naphthalene diimide (NDI), perylene diimide (PDI), and (3*E*,7*E*)‐3,7‐bis(2‐oxoindolin‐3‐ylidene)benzo[1,2‐*b*:4,5‐*b’*]‐difuran‐2,6(3*H*,7*H*)‐dione (IBDF), represent an important class.[^28^, ^29^, ^30^] These building blocks are favored for their electron‐withdrawing capabilities, which facilitate the adjustment of energy levels and optical properties, as well as enhance interchain charge transport. Our recent studies have shown that unsubstituted amide groups on DPP and hemiisoindigo (HID) units within the backbone of π‐conjugated polymers can strongly interact with polar analytes.[^31^, ^32^] Since these unsubstituted amide groups are directly connected to the π‐conjugation system of the polymer backbone, their interactions with analyte molecules effectively interfere with charge carrier transport, resulting in superior response time, sensitivity, and selectivity in chemical sensors.

**Figure 1:**
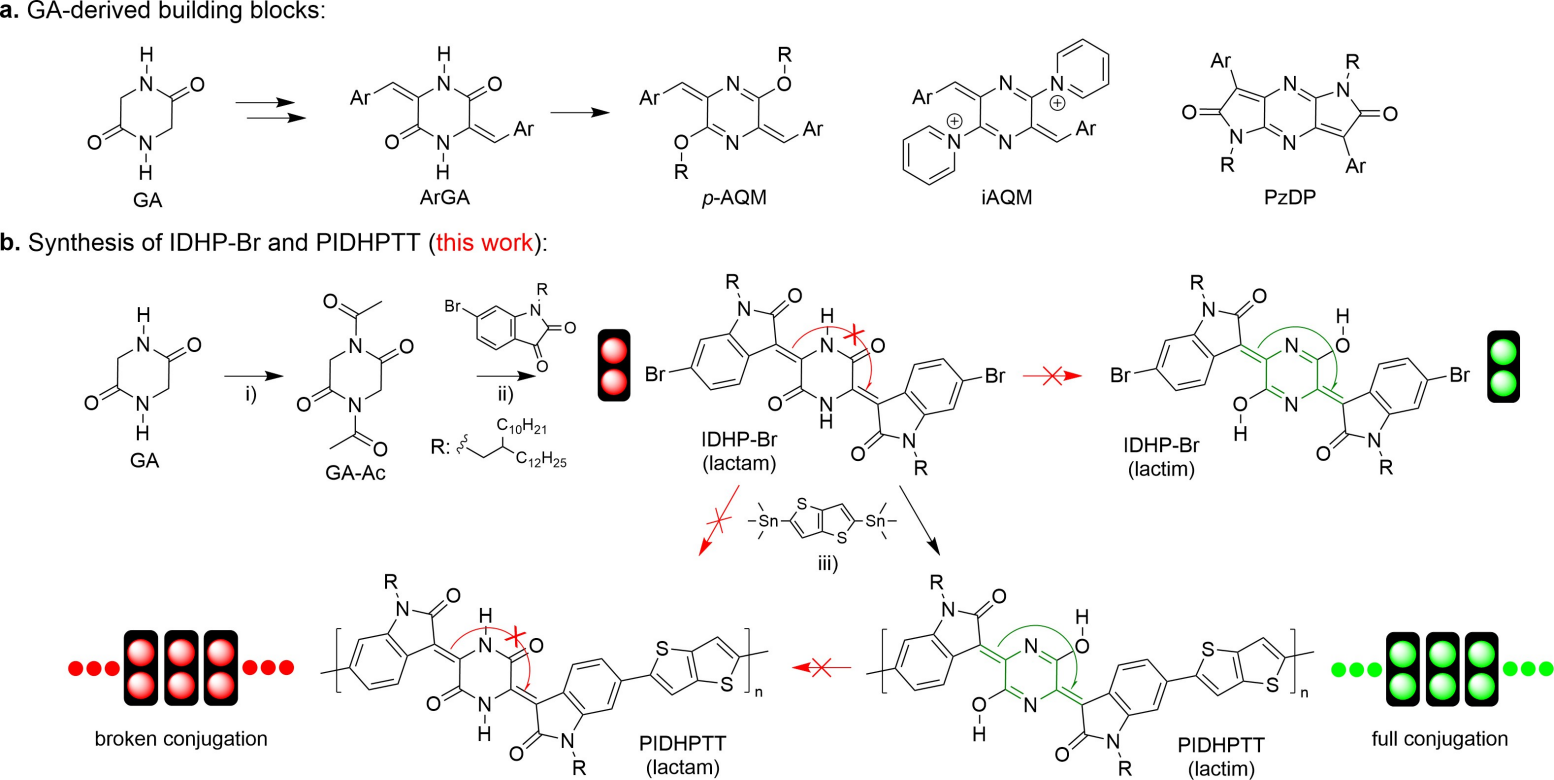
(a) Glycine anhydride (GA)‐derived building blocks, p‐AQM,^[34]^ iAQM,^[36]^ and PzDP,^[33]^ which are used to construct high‐performance π‐conjugated polymer semiconductors; (b) Synthesis of the monomer IDHP−Br (predominantly in its lactam form), comprising the novel GA‐derived building block IDHP, and the polymer PIDHPTT, predominantly in its lactim form, reported in this work: (i) acetic anhydride, reflux, 6 h, 94 % yield; (ii) DMF, Et_3_N, r. t., 6 h, 94 % yield; (iii) Pd_2_dba_3_/P(o‐tol)_3_, chlorobenzene, 110 °C, 48 h, 85 % yield.

Recently, the amide‐based piperazine‐2,5‐dione (glycine anhydride, GA) has been identified as a valuable and readily available precursor building block for constructing high‐performing π‐conjugated polymer semiconductors used in OFETs, sensors, and biomedical applications.[^33^, ^34^, ^35^, ^36^, ^37^, ^38^, ^39^] However, in its lactam form, the GA unit (DHP‐NH) cannot form a conjugation bridge with neighboring π‐conjugated building blocks and has been converted into a quinoidal form to achieve a fully extended conjugation backbone through O‐substitution or other chemical reactions (Figure 1a).

In this work, we developed a novel electron‐acceptor building block, bisindolin‐dihydropiperazine (IDHP), which consists of a GA unit flanked by two isatin units (Figure 1b). This building block was employed to construct a donor‐acceptor (D−A) polymer semiconductor, PIDHPTT, incorporating the well‐known donor building block, thienothiophene (TT). The resulting polymer was used as a water‐stable active layer in water‐gated OFET (WG‐OFET)‐based chemical sensors. The choice of isatin units is based on the excellent stability observed in polymer semiconductors derived from isatin‐containing building blocks such as isoindigo^[25]^ and indigo.^[26]^ More importantly, density functional theory (DFT) simulations indicate that the OH groups in the GA's imidic acid form, 3,6‐dihydropyrazine‐2,5‐diol (lactim DHP‐OH), have much stronger binding energy with fluoride ions compared to the NH groups in the amide form (lactam DHP‐NH). However, the IDHP−Br monomer predominantly adopts the lactam form, as revealed by its NMR and FTIR spectra, indicating broken π‐conjugation in this compound. Surprisingly, once this monomer is incorporated into the polymer PIDHPTT, the GA units are completely self‐converted into the DHP‐OH form, achieving extended full π‐conjugation along the polymer backbone as demonstrated by FTIR and UV/Vis spectroscopic data. DFT simulation results on oligomers show that the lactim tautomer becomes increasingly energetically favorable with the extension of chain length. This trend supports the role of energy minimization in promoting a predominant lactam‐to‐lactim tautomerization. It appears that the presence of a lactim tautomer of GA in the polymer backbone encourages the formation of the lactim form in neighboring repeat units. This cascade formation of the lactim tautomer in GA units resembles the vinylogous effect, where the electronic influence of a functional group is propagated to a remote position through a π‐conjugated pathway.[^40^, ^41^, ^42^] This study is the first to report a long‐range vinylogous effect achieved through the collective synergy of numerous widely spaced functional groups.

The fully conjugated backbone of PIDHPTT facilitates efficient charge transport, while the appropriately positioned frontier energy levels of this D−A polymer enable both hole and electron transport. As a result, when used as a channel semiconductor in OFETs, PIDHPTT exhibited good ambipolar transistor performance with balanced hole and electron mobilities measured at 0.021 cm^2^ V^−1^ s^−1^ and 0.029 cm^2^ V^−1^ s^−1^, respectively. Under p‐channel operation, the WG‐OFET device demonstrated excellent air and aqueous stability, maintaining its electrical properties after soaking in water. We investigated PIDHPTT′s sensing performance toward fluoride ions in aqueous solution, aiming for applications in the analysis and monitoring of fluoride contamination in water sources, where fluoride pollution, which causes adverse health effects, has become a global concern.^[43]^ PIDHPTT exhibited excellent sensitivity and selectivity toward fluoride ions, with a limit of detection (LOD) as low as 0.28 μM. This LOD value is much lower than the WHO recommended fluoride level in drinking water (26.3–78.9 μM), making our sensor devices potentially suitable for detecting fluoride ions. The mechanisms for the selectivity of PIDHPTT for fluoride ions over other halide ions were further investigated through ultraviolet‐visible (UV/Vis) spectroscopy and electrochemical impedance spectroscopy (EIS), which showed dramatically different responses of the polymer to fluoride ions compared to other halide ions. The findings in this work demonstrate that the non‐conjugated lactam GA building block can self‐tautomerize into the fully conjugated lactim form within a polymer backbone through the long‐range vinylogous effect. This makes GA, and potentially other amide‐based compounds, highly promising building blocks for constructing fully conjugated polymer semiconductors for use in chemical sensors and other applications.

## Results and Discussion

### Synthesis and Characterization of PIDHPTT

The IDHP−Br monomer was conveniently prepared by acetylating GA to form 1,4‐diacetylpiperazine‐2,5‐dione (GA−Ac), followed by a Knoevenagel condensation reaction between GA−Ac and 2‐decylteradecyl‐substituted 6‐bromoisatin (6‐bromo‐1‐(2‐decyltetradecyl)indoline‐2,3‐dione) in the presence of triethylamine at room temperature (Figure 1b). PIDHPTT was synthesized through a conventional Stille coupling polymerization between IDHP−Br and 2,5‐bis(trimethylstannyl)‐thienothiophene, with an 85 % isolated yield. The number average molecular weight (*M*_n_), weight average molecular weight (*M*_w_), and dispersity (*Ð*) of PIDHPTT were determined to be 13.1 kDa, 36.0 kDa, and 3.23, respectively, using high‐temperature gel permeation chromatography (HT‐GPC) (Figure S13).

**Figure 2:**
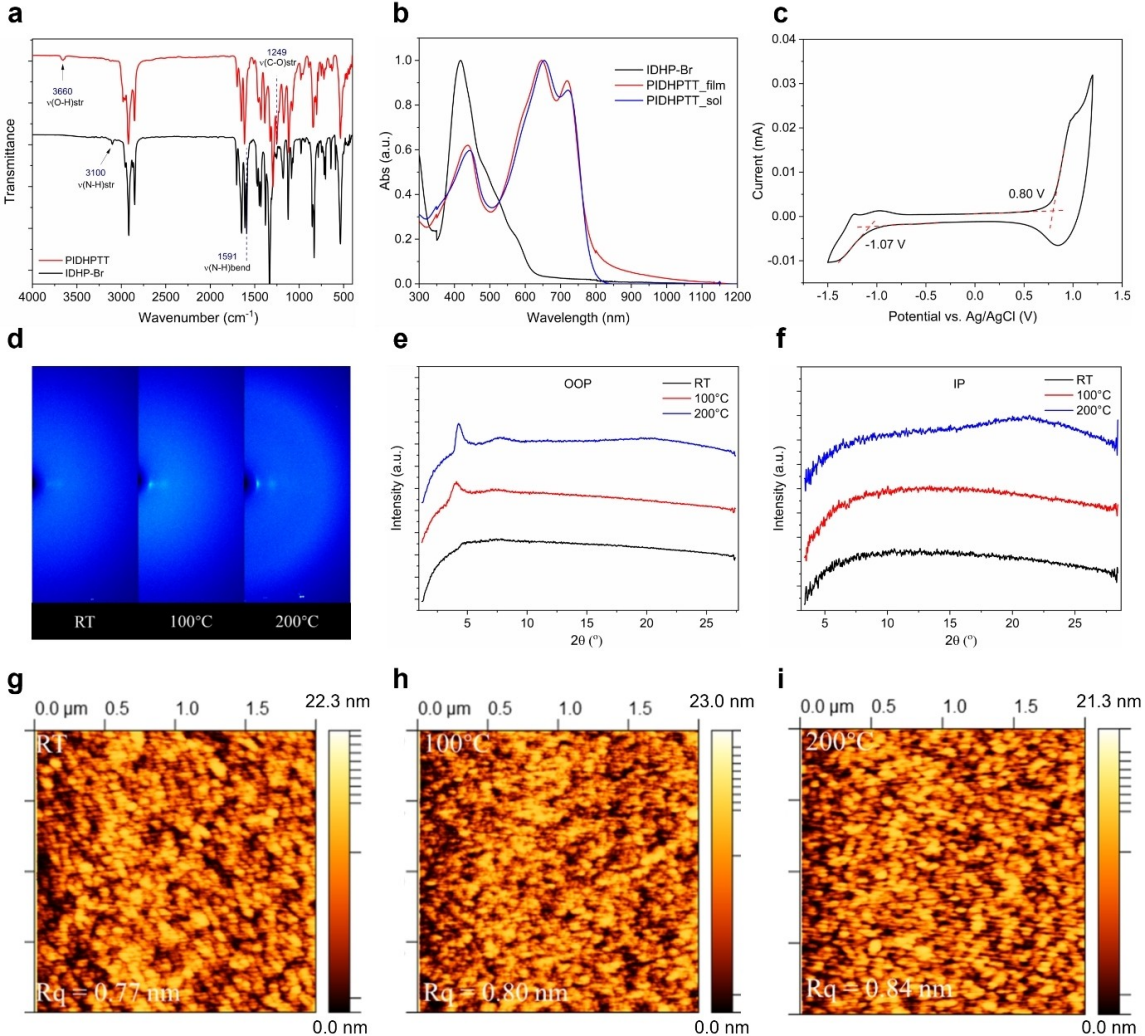
(a) FTIR transmittance spectrum of IDHP−Br monomer and PIDHPTT polymer. (b) UV/Vis absorption spectrum of IDHP−Br monomer and PIDHPTT polymer in solution (chloroform) and thin film state. (c) Cyclic voltammogram of PIDHPTT film. (d) 2D‐GIXD scattering pattern for PIDHPTT thin film annealed at different temperatures, with line‐cut profiles in the (e) out‐of‐plane (OOP) and (f) in‐plane (IP) directions. (g)–(i) AFM images of PIDHPTT thin films without annealing (RT) and annealed at 100 and 200 °C.

The ^1^H spectrum of IDHP−Br shows the characteristic N−H peak at 13.37 ppm (Figure S3), while its FTIR spectrum exhibits distinctive N−H stretching (υ=3100 cm^−1^) and N−H bending (υ=1591 cm^−1^) vibrations (Figure 2a, S2), indicating the predominant presence of the lactam form of the DHP unit in IDHP−Br.

On the other hand, the FTIR spectrum of the polymer PIDHPTT no longer exhibits the two NH vibrations, while a peak representing OH stretching (υ=3660 cm^−1^) appears. Additionally, a vibration peak at 1249 cm^−1^, absent in IDHP−Br, also appears and is assigned to the C−OH stretching in the lactim form of IDHP, as C‐OH stretching from alcohols/phenols typically falls within the range of 1050–1250 cm^−1^.[^44^, ^45^] Due to strong chain aggregation of PIDHPTT in solvents, which resulted in broadening of the signals, attempts to detect the disappearance of N−H groups in PIDHPTT using NMR were unsuccessful, even at elevated temperatures in deuterated tetrachloroethane (Figure S5). Nonetheless, the FTIR results unequivocally indicate that the central DHP rings in the repeat units of PIDHPTT have completely switched to the lactim form from the lactam form present in the monomer IDHP−Br. As mentioned earlier, the quinoidal lactim form of DHP functions as a π‐conjugated bridge, connecting the neighboring isatin units to realize a fully conjugated polymer backbone, which is beneficial for efficient charge transport along the polymer main chain.

UV/Vis spectroscopy analysis was conducted to investigate the optical properties of PIDHPTT. As shown in Figure 2b, PIDHPTT in solution exhibits two strong low‐energy absorption peaks at 654 and 721 nm, along with a high‐energy absorption peak at 443 nm. In the film, all three absorption bands slightly blue‐shift to 439, 647, and 718 nm compared to the solution spectrum, likely due to the formation of H‐aggregates in the solid state.[^46^, ^47^] The onset wavelength (λ_onset_) for the PIDHPTT thin film is 789 nm, corresponding to a small optical band gap of 1.57 eV. Compared to the monomer IDHP−Br, PIDHPTT exhibits a red shift in the wavelength of the maximum absorption band (λ_max_=718 nm for PIDHPTT and λ_max_=417 nm for IDHP−Br). This supports the extended delocalization of π‐electrons along the polymer backbone in PIDHPTT, facilitated by the formation of the lactim form of the DHP units as discussed earlier.

Cyclic voltammetry (CV) measurements were performed on the PIDHPTT film to determine its frontier molecular orbital energy levels. An Ag/AgCl electrode was used as a reference electrode, which is further referenced with ferrocene (Figure S15) that has a known oxidative potential of −4.80 eV.^[48]^ The CV diagram of PIDHPTT showed one sharp peak for the oxidation curve while the reduction curve is quite broad and lower in intensity. The corresponding highest occupied molecular orbital (HOMO) and lowest unoccupied molecular orbital (LUMO) energy levels were estimated to be −5.58 eV and −3.71 eV, from the oxidative and reductive onset potentials, respectively. The electrochemical band gap is 1.87 eV, which is larger than the optical band gap (1.57 eV), due to the notable exciton binding energy commonly observed for organic semiconductors.[^49^, ^50^, ^51^]

The crystallinity of PIDHPTT thin films was measured using two‐dimensional grazing incidence X‐ray diffraction (2D‐GIXD) spectroscopy (Figure 2d). Figures 2e and 2 f show the in‐plane (IP) and out‐of‐plane (OOP) X‐ray diffraction patterns of PIDHPTT thin films annealed at different temperatures. In both directions, the as‐spun (room temperature, RT) polymer film shows no obvious diffraction peak, indicating the amorphous nature of this polymer without annealing. After annealing at 100 °C, the polymer thin film shows a peak at 2θ=4.05°, corresponding to a d‐spacing of 21.8 Å, which can be assigned to the packing distance between the (100) planes of a lamellar crystal structure. The absence of a noticeable peak in the IP direction suggests that the polymer thin film adopted an edge‐on dominant orientation. Further annealing of the polymer thin film at 200 °C resulted in a shift of the (100) peak to 2θ=4.26° (d‐spacing=20.7 Å) in the OOP direction, indicating tighter packing of the lamellar layers. The peak also exhibits a sharper shape and higher intensity compared to the thin film annealed at 100 °C, indicating improved crystallinity with higher annealing temperatures. Additionally, the improved crystallinity is supported by the appearance of a broad hump centered at 2θ≈21.4° (d‐spacing of 0.42 nm) in the IP direction, which corresponds to the (010) diffraction peak. This indicates increased order along the π–π stacking direction, further confirming the enhanced crystallinity with thermal annealing. However, the π–π stacking distance (0.42 nm) is larger than that typically observed in high‐mobility polymer semiconductors, where it is usually less than 0.38 nm.^[52]^ The increased distance is caused by the twisting of the polymer backbone due to the steric effect of the neighboring isatin units on the DHP unit, which would hinder interchain charge transport.

The morphology of the thin film samples annealed at different temperatures was examined using atomic force microscopy (AFM). As shown in Figure 2g, the as‐spun (RT) film is composed of tightly packed small grains, tens of nanometers in size, with clear grain boundaries and a small root mean square roughness (Rq) of 0.77 nm. After annealing at 100 °C and 200 °C, the morphology of the films remains similar, with slight increases in Rq to 0.80 nm and 0.84 nm, respectively (Figures 2h and 2i). These increases in roughness may be attributed to the increased crystallinity of the films with higher annealing temperatures. While the tight connection of grains ensures efficient charge transport, the presence of evenly distributed nanometer‐sized grain boundaries facilitates the diffusion of analyte species into the polymer, thereby enhancing sensitivity.

### Theoretical Calculations

Lactam‐lactim tautomerization is influenced by external factors such as light, temperature, solvent, pH, and salt as well as internal factors such as stability of functional groups, push‐pull effect, intramolecular H‐bonding, and aromaticity.[^53^, ^54^, ^55^, ^56^, ^57^, ^58^, ^59^, ^60^, ^61^, ^62^] Since the FTIR spectra of both IDHP−Br and PIDHPTT were acquired in their solid states, the observed complete lactam‐to‐lactim conversion of DHP units in PIDHPTT is considered to be attributed to the internal factor of extended chain length, specifically the effect of π‐electron delocalization.^[56]^ To gain insights into the predominant formation of the lactim form of the DHP units in PIDHPTT, DFT calculations were performed on the monomer IDHP−Br and on oligomers of PIDHPTT with varying backbone lengths using Gaussian 16 software with B3LYP/6‐31G(d) basis set.

**Figure 3:**
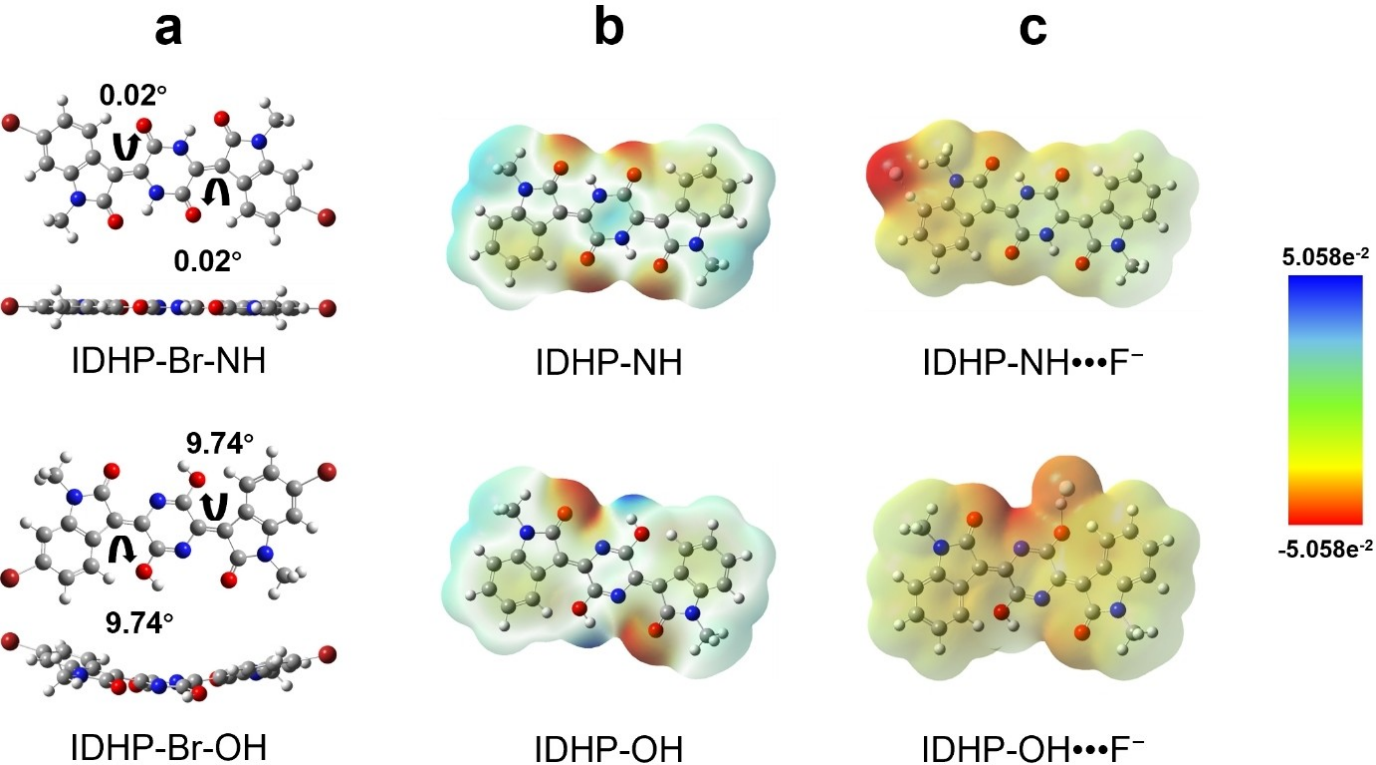
DFT‐simulation results: (a) Molecular geometries of IDHP−Br‐NH (lactam) and IDHP‐OH (lactim); (b) The electrostatic potential (ESP) surfaces of IDHP‐NH and IDHP‐OH, where the negative and positive charges are represented by red and blue colors, respectively; (c) The ESP surfaces of energy‐optimized IDHP‐NH•••F^−^ and IDHP‐OH•••F^−^ species.

Figure 3 shows the DFT simulation results for IDHP−Br in both its lactam and lactim forms. By comparing the total energies, it appears that the lactam form is more stable than the lactim form by 184.05 kJ mol^−1^. The lower energy of the lactam IDHP−Br is likely due to the more preferred lactam form of the DHP unit as observed for most other lactam‐lactim systems in their pristine state.[^56^, ^63^] Additionally, the lactam form is highly coplanar with very small dihedral angles (0.02°) compared to the lactim form, which is twisted and bent, with larger dihedral angles (9.74°), due to the steric hindrance between the isatin units and the DHP ring. These simulation results agree with the FTIR data that shows the predominant lactam form of IDHP−Br.

**Figure 4:**
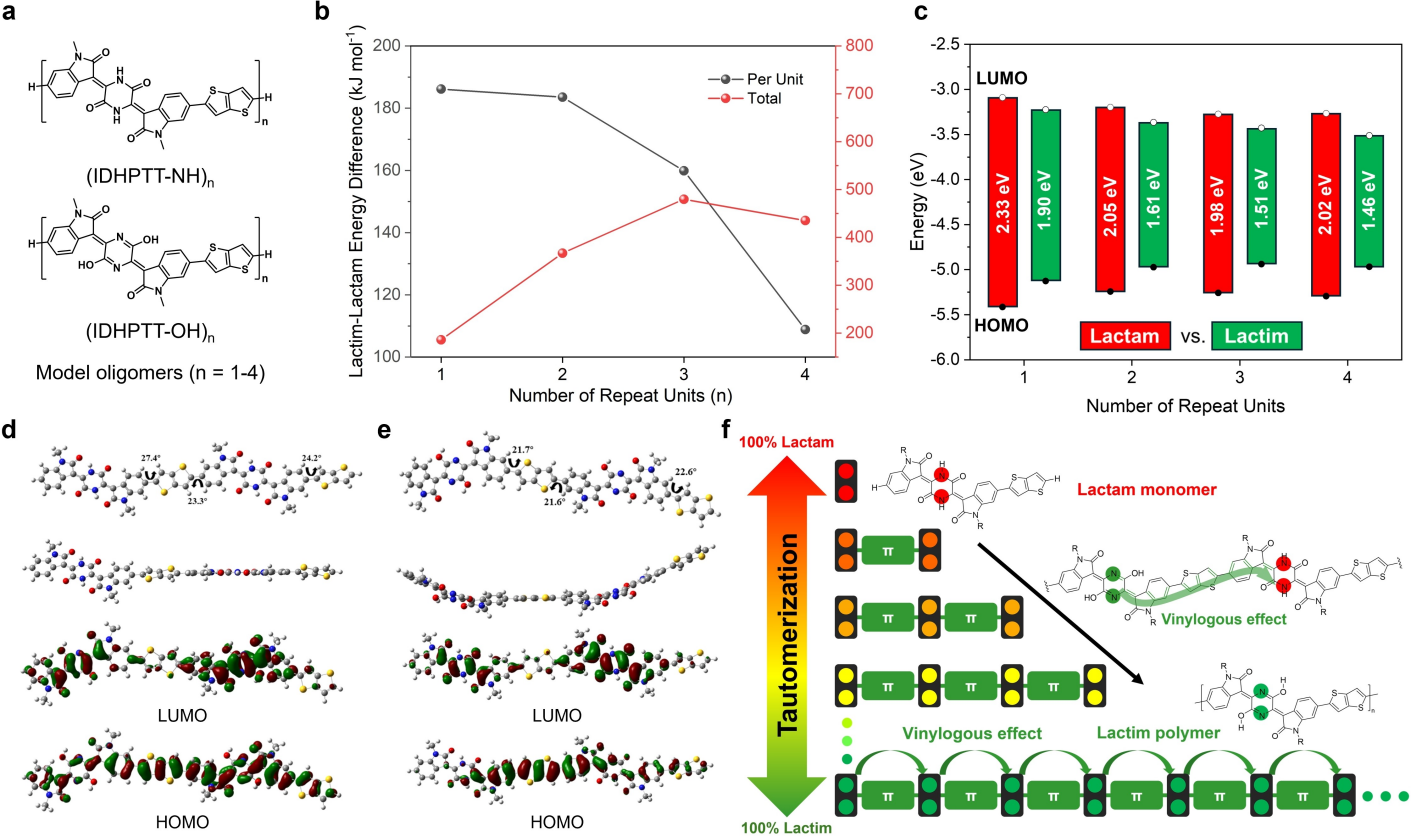
(a) Chemical structures of oligomers in lactam and lactim forms for PIDHPTT used in DFT simulations, with methyl groups substituted for 2‐decyltetradecyl chains to expedite computation. (b) Energy differences between oligomers in their lactam and lactim forms obtained from DFT simulations. The dashed lines represent the energy difference as a function of the number of repeat units, assuming no energy minimization by the vinylogous effect from neighboring units in the lactim form. (c) Energy levels and band gaps of oligomers obtained from DFT simulations. (d) Optimized geometries, HOMO, and LUMO of the lactam dimer, DIDHP‐NH. (e) Optimized geometries, HOMO, and LUMO of the lactim dimer, DIDHP‐OH. (f) Schematic illustration of the vinylogous effect promoting tautomerization from the predominant lactam monomer to the predominant lactim polymer with increasing chain length.

Next, we simulated the monomer and oligomers of PIDHPTT, with up to 4 repeat units, in both their lactam and lactim forms (Figure 4 and Table S2). The lactim form monomer (IDHPTT‐OH) has a total energy higher by 184.57 kJ mol^−1^ than the lactam form (IDHPTT‐NH), which is slightly higher than that between the two IDHP−Br tautomers (184.05 kJ mol^−1^), indicating that the lactam form is still energetically preferred.

As shown in Figures 4d and 4e, the IDHP units remain twisted and bent in the lactim dimer (DIDHPTT‐OH) and coplanar in the corresponding lactam form (DIDHPTT‐NH), similar to the monomeric tautomers. However, the dihedral angles between an isatin unit and a TT unit in the lactim form are smaller, ranging from 21 to 22°, compared to 23–27° in the lactam form. This smaller angle in the lactim form is advantageous for more effective π‐bridging between the two isatin units. This effect can be more clearly seen in a dramatic reduction in the band gap to 1.61 eV for the lactim dimer from a value of 1.90 eV for the lactim monomer (Figure 4c). Extended delocalization of π‐electrons along the longer backbone is expected to contribute to the stabilization of the lactim dimer compared to the lactim monomer. Consequently, although the total energy of the lactim form dimer is still higher than the lactam form by 367.04 kJ mol^−1^, the energy difference between the two forms slightly decreased to 183.52 kJ mol^−1^ per repeat unit compared to that of the monomer (184.57 kJ mol^−1^). Notably, the band gap of the lactam dimer decreases to 2.05 eV compared to 2.33 eV for the lactam monomer. This reduction is attributed to the bridging of two isatin units by a TT unit in the lactam dimer, which forms a longer conjugation segment, isatin‐TT‐isatin.

Simulation results for the trimer indicate a substantial reduction in energy difference per repeat unit between the two tautomers, down to 159.89 kJ mol^−1^. For the tetramer, the energy difference between the lactim and lactam forms decreased even further, reaching 108.83 kJ mol^−1^ (Figure 4b and Table S2). Additionally, the total energy difference between the two tetramer tautomers is smaller compared to that of the trimers (Figure 4b).

Furthermore, the C−N bond lengths in the DHP units of the lactam tautomers gradually increase from the monomer to the tetramer, while the C−O bond lengths in the DHP units of the lactim tautomers decrease progressively starting from the dimer (Table S2). These results reflect the enhanced effect of π‐electron delocalization on energy minimization in the lactim tetramer compared to the shorter oligomers. As shown in Figure 4c, the band gaps of the lactam trimer and tetramer are similar to those of the lactam dimer. The non‐conjugated lactam DHP units impede π‐electron delocalization between the conjugated segments (isatin‐TT‐isatin), resulting in minimal change in the effective conjugation length. In stark contrast, the lactim trimer and tetramer exhibit progressively smaller band gaps of 1.51 eV and 1.46 eV, respectively, indicating an extension of the effective conjugation length with an increasing number of repeat units. Due to limitations in DFT computation, oligomers with more than four repeat units were unsuccessful.

To support the gradual tautomerization from lactam to lactim with increasing repeat units, monomer (IDHP‐2T, equivalent to IDHPTT), dimer (IDHPTT‐dimer), and tetramer (IDHPTT‐tetramer) model compounds with thiophene end groups were synthesized (Scheme S1). ^1^H NMR analysis revealed that IDHP‐2T and IDHPTT‐dimer contained 86 % and 59 % of N−H groups (lactam form), respectively, while the comonomer IDHP−Br, with a shorter conjugation length, had 96 % lactim (Figure S11). This gradual tautomerization was further supported by FTIR, which showed a weakening of the N−H vibration (Figure S2). For IDHPTT‐tetramer, ^1^H NMR signals were unresolved even at elevated temperatures, similar to the polymer PIDHPTT. However, its FTIR spectrum showed a further reduction in the N−H peak intensity, confirming increased lactam‐to‐lactim conversion. UV/Vis spectra of these oligomers (Figure S1) demonstrated a decreasing band gap with increasing backbone length, reflecting enhanced delocalization of the conjugated system due to lactim bridge formation. These findings align with DFT simulation results.

These results manifest the significant effect of π‐electron delocalization on the energy minimization of the polymer, as schematically illustrated in Figure 4f. Since the number of repeat units in PIDHPTT is about 11 based on its *M*_n_, it is expected that the lactim form would become more energetically stable than the lactam form. It appears that the presence of the lactim form of a DHP unit promotes the formation of the lactim form in neighboring DHP units through the extended π‐conjugated isatin‐TT‐isatin linkages between them in the backbone of PIDHPTT. This phenomenon is reminiscent of the vinylogous effect observed in some π‐conjugated systems, where the electronic influence of a functional group on one end of the conjugated system is transmitted to the remote functional group at the other end.[^40^, ^41^, ^42^] To the best of our knowledge, this is the first report on such a long‐range vinylogous effect in a polymer and the first to utilize the vinylogous effect to achieve fully extended polymer backbone π‐conjugation through self‐tautomerization.

Electrostatic potential (ESP) surface simulations using the DFT method were performed to identify potential sites to bind a halide ion on two IDHP tautomers, lactam IDHP‐NH and lactim IDHP‐OH. It appears that in IDHP‐NH the positively charged proton in each N−H group is guarded by two nearby electron‐rich carbonyl groups (Figure 3b). Consequently, accessing an N−H site by a fluoride ion is thermodynamically unfavorable. The simulation indicates that fluoride would instead position next to a benzylic proton and a proton of a methyl substituent on the isatin moiety. After fluoride binding, the ESP surface for IDHP‐NH shows high electron density due to weak interaction between the fluoride and the aromatic proton. The interatomic distance for the F•••H hydrogen bond is 1.53 Å.

In contrast, each OH group in pristine IDHP‐OH is adjacent to only one electron‐rich carbonyl group, with the positively charged proton on the OH group fully exposed at the molecule's periphery, making it accessible to fluoride ions. As shown in Figure 3c and Figure S16, fluoride preferentially binds to the OH group in IDHP‐OH, as evidenced by a reduced electron density on the fluoride ion, a much shorter interatomic F•••H distance (0.97 Å), and a significantly larger binding energy (EB) (−452.37 kJ mol^−1^) compared to IDHP‐NH (−281.98 kJ mol^−1^). These simulation results indicate that the presence of the lactim IDHP‐OH in PIDHPTT is more favorable for achieving high sensitivity toward fluoride ions.

Additionally, chloride binding with IDHP‐OH was also simulated, revealing a longer interatomic Cl•••H distance (1.74 Å) and a smaller binding energy (−116.49 kJ mol^−1^) (Figure S16), indicating that PIDHPTT has a lower affinity for chloride ions compared to fluoride ions.

Interestingly, DFT simulations indicate that a bromide ion cannot access the OH group in IDHP‐OH due to repulsion from the nearby carbonyl group, as the bromide ion has a much larger electron cloud compared to chloride and fluoride ions (Figure S16). As a result, the bromide ion positions next to the benzylic proton, similar to the binding of the fluoride ion with IDHP‐NH. Optimal molecular geometry, ESP surfaces, binding energy, and interatomic distances could not be determined for bromide binding simulations (Figure S16d). Nevertheless, these simulation results suggest a binding affinity order of F^−^ > Cl^−^ > Br^−^, which is beneficial for achieving good selectivity when integrating PIDHPTT as a functional material in an OFET sensor.[^64^, ^65^]

### SiO_2_‐Gated Organic Field‐Effect Transistor (OFET) Performance

**Figure 5:**
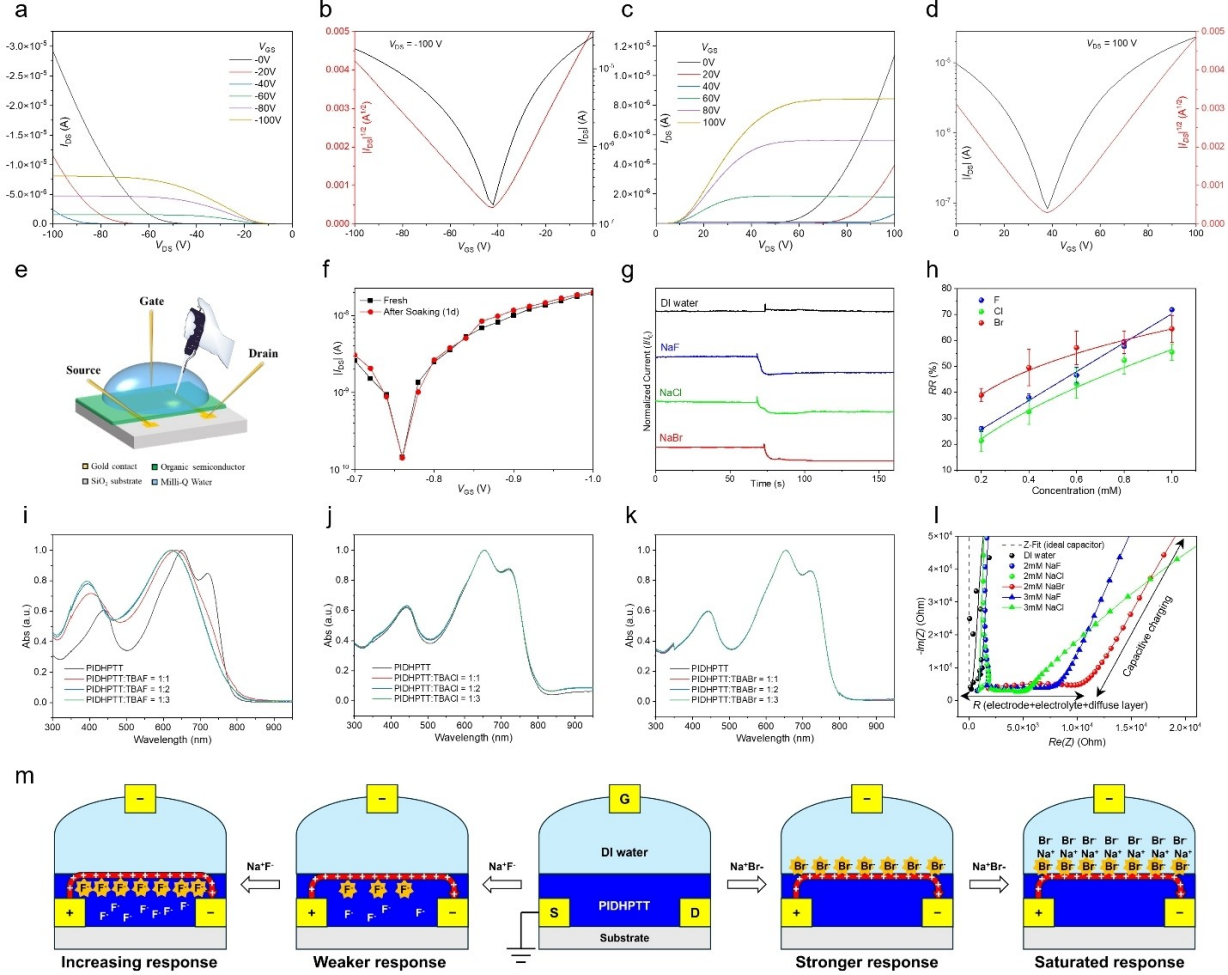
(a) Output and (b) transfer curves in p‐channel operation, and (c) output and (d) transfer curves in n‐channel operation for a representative BGBC OFET device based on PIDHPTT (annealed at 250 °C) fabricated on an n^+^‐Si/SiO_2_ substrate. (e) Schematic illustration of the water‐gated OFET sensor device for halide ion sensing. (f) P‐channel transfer characteristics (*V*_DS_=−0.5 V) of a freshly fabricated device compared to the same device after soaking in water for one day. (g) *I*_DS_ versus time profiles under constant *V*_DS_ of −0.5 V and *V*_GS_ of −1 V, where 5 μL of pure DI water or a sodium halide solution was injected after about 60 seconds. The concentration of the injected sodium halide solution is 5 mM, resulting in a sodium halide concentration of 1 mM in the water gate dielectric. h) *RR* of sensors to different halide ions at varying concentrations (average values from five devices per data point). (i)–(k) UV/Vis spectra of PIDHPTT solutions in chloroform before and after the addition of 1, 2, and 3 molar equivalents of TBAF, TBACl, and TBABr, respectively. (l) Nyquist profiles with 2 and 3 mM of fluoride and chloride ions, and 2 mM of bromide ions obtained from electrochemical impedance spectroscopy (EIS) measurements, scanning from 1000 kHz to 100 mHz under 500 mV. (m) Proposed sensing mechanisms of PIDHPTT‐based WG‐OFET sensors for fluoride and bromide ions.

To investigate the transistor performance of PIDHPTT, the polymer was used as a channel material in OFETs with a bottom‐gate bottom‐contact (BGBC) device geometry using a heavily n‐dope silicon wafer with a thermally grown SiO_2_ layer as the dielectric. The devices were characterized inside a glove box filled with nitrogen. As shown in Figures 5a–d and Table S3, transfer and output curves of PIDHPTT exhibited typical ambipolar performance with balanced hole (*μ*_h_) and electron (*μ*_e_) mobilities, attributed to the favorable HOMO and LUMO energy levels for both hole and electron transport. Both hole and electron mobilities increased with thermal annealing due to the improved crystallinity of the polymer, as revealed by the XRD data. Saturation *μ*_h_ and *μ*_e_ reach up to 0.0245 cm^2^ V^−1^ s^−1^ and 0.0349 cm^2^ V^−1^ s^−1^, respectively, at an annealing temperature of 250 °C. The twisted structure of the IDHP units, as shown by DFT simulations, hampers charge transport. Reducing the steric effects of neighboring isatin units on DHP could enhance backbone coplanarity, potentially shortening the π–π stacking distance and improving both interchain and intrachain charge transport.

The threshold voltages (*V*_T_) for both p‐type and n‐type operations are rather high, indicating the presence of a significant number of hole and electron traps. Thermal annealing up to 200 °C can effectively diminish the number of hole traps, resulting in much decreased p‐channel *V*_T_. In contrast, electron trap densities are not affected by thermal annealing as no obvious change in n‐channel *V*_T_ is observed with increasing annealing temperature. Further increasing the annealing temperature to 250 °C causes a slight relapse of *V*_T_ for both channels, likely due to the delamination between polymer film and silicon substrate under high annealing temperatures.^[66]^ Beyond an annealing temperature of 100 °C, the devices suffer from a high level of off‐current, resulting in relatively low on/off current ratios (*I*_on/_ *I*_off_). This phenomenon is typical for intrinsic ambipolar materials as a gate electrode is incapable of depleting a large number of holes and electrons present in the ambipolar channel simultaneously.[^67^, ^68^]

### Halide Ion Sensing with WG‐OFET's

Fluoride ion sensing experiments were conducted for PIDHPTT in a WG‐OFET device fabricated on Si/SiO_2_ wafer substrates. The sensor device has an interdigitated gold source and drain electrodes with a channel length of 30 μm and a channel width of 15.8 mm. For sensing measurements, 20 μL of Milli‐Q deionized (DI) water with a resistivity of 18 MΩ⋅cm was placed on top of the sensors as a dielectric to fully cover the source and drain electrodes. A probe needle was positioned above the polymer film and in contact with the water dielectric, serving as a floating gate (Figure 5e). To prevent electrolysis of water, which may start to occur at a potential difference of 1.23 V,^[69]^ the absolute values of *V*_GS_ and *V*_DS_ were kept below 1 V during operation. The devices could not function in n‐channel mode in ambient air and water because the LUMO energy level of PIDHPTT (−3.71 eV) is higher than the required maximum level of approximately −4.0 eV for stable electron transport in these conditions.[^70^, ^71^] Therefore, only the p‐channel mode was used for the sensing experiments. Figure 5f shows the transfer characteristics of the device at *V*_DS_=−0.5 V, indicating typical p‐type transistor behavior. The device demonstrated stable operation in ambient air and showed no variation in transfer characteristics observed after soaking the device in water for one day. The device's excellent stability in water is primarily due to PIDHPTT's favorable HOMO energy level (−5.58 eV), which falls within the required range of −5.0 eV to −5.6 eV for stable hole transport.^[52]^ Additionally, the water resistance of the hydrophobic long alkyl side chains of the polymer contributes to its morphological stability.

To study the sensing performance of PIDHPTT‐based WG‐OFET devices toward halide ions, aqueous sodium halide solutions (NaF, NaCl, and NaBr) with various concentrations were used as analytes. As shown in Figure 5g, the device was first operated at *V*_DS_=−0.5 V and *V*_GS_=−1.0 V with 20 μL of DI water as dielectric for approximately 60 s to ensure a stable *I_DS_* as the baseline. Then, 5 μL of DI water (blank) or a sodium halide stock solution was injected into the water dielectric. Injection of DI water induced no change in *I*_DS_ after the device was stabilized, while injection of a halide solution caused a drop in *I*_DS_.

The sensing performance of the sensor devices towards halide ions was characterized by calculating the relative response (*RR*), $RR=\left(\left|\Delta I\right|/\left|I_{0}\right|\right)\times 100\;\%$ , where *I_0_* is the baseline *I_DS_* and ΔI is the change in *I_DS_* after analyte addition.[^72^, ^73^, ^74^] Figure 5h shows the linear regression fit for the *RR* vs. NaF concentration data points in the range of 0–1.0 mM. The slope (*m*) and coefficient of determination (R^2^) are 65.9 and 0.992, respectively. The limit of detection (LOD) of the sensor was calculated using the equation: LOD=3.3σ/m , where *m* is the slope of the calibration (*RR* versus analyte concentration) curve, and *σ* is the relative standard deviation of the sensitivity plot for the blank sample.^[75]^ The LOD of the sensor to sodium fluoride was thus calculated as 0.28 μM. Both the sensitivity and LOD outperformed our previously reported indigo‐based WG‐OFET fluoride sensor.^[73]^ Although the LOD of our sensor is higher than that of most state‐of‐the‐art colorimetric and fluorescence‐based aqueous fluoride sensors, which detect fluoride ions at the nanomolar level,[^76^, ^77^, ^78^] our sensor offers a simpler fabrication process, lower costs, lighter weight, and efficient chemical‐to‐electrical signal conversion. These advantages make our sensor a practical, cost‐effective alternative for fluoride detection in drinking water and other applications. The sensor devices can be recovered by rinsing the polymer film with deionized water and reused multiple times without noticeable performance degradation.

As shown in Figure 5h, the device exhibits lower *RR* values to NaCl compared to NaF at the same concentrations and a deviation of *RR* from the linear fit when the chloride concentration exceeds 0.6 mM, indicating the existence of an equilibrium state for the absorption of chloride ion.[^79^, ^80^, ^81^, ^82^] The sensing profiles for fluoride and chloride ions align with our simulation results, which indicate stronger binding of fluoride ions with PIDHPTT compared to chloride ions.

Unexpectedly, the device exhibits a higher *RR* to NaBr at a low concentration of 0.2 mM compared to NaF and NaCl, despite DFT simulation results suggesting that bromide binding to PIDHPTT is unlikely. Additionally, compared to NaF and NaCl, the *RR* increases at a much slower rate with increasing NaBr concentration, indicating that the sensor becomes saturated much earlier.

To further investigate the effect of halide ions on the electrical properties of PIDHPTT, transfer curves of the sensors were measured at different halide concentrations (Figure S17). An obvious increase in *V*_T_ (absolute value) is observed with increasing fluoride concentration, indicating a higher hole trap density introduced at a higher fluoride concentration. For chloride ion sensing, the *V*_T_ shifts more significantly at 0.2 mM but changes at a slower rate with increasing chloride concentration compared to fluoride. In the case of bromide ion sensing, the largest increase in *V*_T_ is observed among the three types of halides, while the change in *V*_T_ remains almost constant with further increasing bromide concentration. As clearly seen in Figures S17a‐c, the shift in *V*_T_ results in a reduction in *I*_DS_ at *V*_DS_=−1 V, corresponding to a decrease in *I*_DS_ observed during the static sensing tests, where *V*_GS_ and *V*_DS_ were held constant at −1 V and −0.5 V, respectively (Figure 5g). As shown in Figure S17d, the changes in *V*_T_ with the halide concentration resemble the changes in *RR* with the halide concentration measured in the static sensing tests shown in Figure 5g. These results suggest that halide ions act as hole traps, leading to a decrease in carrier density in the channel and a reduction in *I*_DS_.

### Study of Halide Ion Sensing Mechanisms Using UV/Vis Spectroscopy and Electrochemical Impedance Spectroscopy (EIS)

UV/Vis spectroscopy was employed to investigate the interaction between PIDHPTT and various halide ions. Tetrabutylammonium (TBA) halide salts (TBAF, TBACl, and TBABr) were used to introduce 1–3 molar equivalents of halide ions relative to the polymer repeat units into PIDHPTT solutions. The addition of one molar equivalent of fluoride ions causes a significant blue shift in the absorption profile of PIDHPTT (Figure 5i). Adding two molar equivalents of fluoride ions results in a slight additional blue shift, while the introduction of three molar equivalents of fluoride ions does not produce further changes in the absorption profile. These observations are in excellent agreement with the DFT simulation results, which show the strong binding of a fluoride ion with the OH group in IDHP through hydrogen bonding (Figure 3c). The binding of fluoride ions reduces the electron‐withdrawing nature of the electron‐accepting IDHP unit, disrupting the donor‐to‐acceptor intramolecular charge transfer. This results in a blue shift of the longest wavelength absorption peak associated with the charge transfer process. During the p‐channel operation of the sensor, fluoride ions bound to the IDHP units would trap holes,[^83^, ^84^] resulting in an increase *V*_T_ and consequently a drop in *I*_DS_.

In comparison, the addition of chloride ions causes only subtle changes in the absorption profile (Figure 5j), indicating a much weaker interaction with the polymer and a lesser impact on its electronic structure, as revealed by the DFT simulation results. Therefore, the device exhibited lower sensitivity towards chloride ions. For the polymer solutions with the addition of bromide ions, almost no noticeable change is observed (Figure 5k), consistent with the DFT prediction that the interaction of bromide ions with the IDHP units is extremely weak. In this case, it is likely that the unbound bromide ions accumulate on top of the polymer film, impeding hole transport in the active layer, which contributes to the high sensitivity of the device towards bromide ions even at low concentrations.

To further investigate the interaction between halide ions and PIDHPTT in the sensor setting, the sensor device was subjected to EIS measurements. Specifically, an alternating current (AC) voltage was applied between the source and gate electrodes, with either pure DI water or an aqueous sodium halide solution covering the polymer film and connecting the electrodes. If halide ions are fully absorbed by the polymer film, no mobile ions would be available, and the device would behave as a capacitor. Conversely, if halide ions are not fully absorbed by the polymer film, ion migration between the two electrodes would occur, and the device would function as an electrolyte cell.[^85^, ^86^]

As shown in Figure 5l, the Nyquist plot of the device with DI water displays a vertical line originating near the origin, indicating typical capacitive behavior. At 1 mM and 2 mM concentrations, the Nyquist plots for NaF and NaCl solutions also exhibit a capacitive Z‐fit, suggesting the absorption of all fluoride or chloride ions by the polymer film (Figure 5l and Figure S18). However, increasing the concentration of fluoride and chloride ions to 3 mM results in behavior resembling that of an electrolyte cell.[^85^, ^86^] This is characterized by a large real impedance due to the resistance of the electrode, electrolyte, and diffuse layer, followed by the appearance of a sloped straight line at about 100 kHz, indicating capacitive charging as the scanning frequency decreases. This suggests that the polymer film is saturated with fluoride or chloride ions and mobile ions are present in the solution at this concentration, aligning with the observed saturation of the sensor's *RR* when the concentration of fluoride or chloride exceeds 2 mM (Figure S19).

In contrast, the plots measured with NaBr solutions at 1 mM (Figure S18b) and 2 mM (Figure 5l) are characteristic of an electrolyte cell, indicating the presence of free bromide ions in the solution. In other words, bromide ions interact poorly with or are not absorbed by the polymer film, which is consistent with the DFT simulation and UV/Vis data. During the WG‐OFET sensing test, the free bromide ions injected into the water dielectric are expected to immediately accumulate on the surface of the polymer channel layer near the source electrode under DC (direct current) negative *V*_GS_ and *V*_DS_ (Figure 5m). The negatively charged bromide ions act as traps for the holes in the active layer, which is located near the top surface of the polymer film. At lower concentrations (e.g., 0.2 mM), all bromide ions are accumulated on the surface of the polymer layer to form a Stern layer. Since the holes are confined to the first few layers of edge‐on oriented organic semiconductor molecules in the channel,[^87^, ^88^] the accumulated bromide ions interact with and trap holes, causing a shift in *V*_T_ and a drop in *I*_DS_. However, because the available surface area of the polymer film is limited, it becomes quickly saturated with bromide ions as the concentration increases. Along with the sodium cations, the excess bromide ions stack on the Stern layer, forming an electric double layer,^[85]^ which exerts minimal impact on hole transport within the active layer. As a result, the device exhibits a stabilized *V*_T_ (Figure S17d) and a saturation in *RR* (Figure 5h).

Conversely, fluoride ions can be strongly absorbed by the entire polymer film, resulting in fewer fluoride ions in the active layer at lower concentrations (<0.8 mM) or fewer hole traps compared to bromide ions. This leads to a smaller *V*_T_ shift and a weaker *RR* (Figure 5m). Since fluoride ions are absorbed uniformly throughout the polymer film, the sensor exhibits a linear relationship between the response and fluoride concentration below saturation concentration (~1–2 mM).

Chloride ions exhibit behavior intermediate between fluoride and bromide ions: they can be absorbed by the polymer film like fluoride ions, but their weak interaction with the polymer leads to surface accumulation, similar to bromide ions. This results in moderate sensor sensitivity and an earlier onset of saturation in *RR* compared to fluoride ions.

## Conclusion

In this work, we introduced PIDHPTT, a novel water‐stable, fully π‐conjugated polymer derived from the non‐conjugated building block glycine anhydride (GA). Through FTIR and UV/Vis analyses, we confirmed that the GA unit within the isatin‐flanked monomer, IDHP−Br, undergoes a complete lactam‐to‐lactim transformation upon polymerization, resulting in fully extended π‐conjugation along the polymer backbone. This tautomerization, driven by energy minimization and facilitated by neighboring DHP units in their lactam form, demonstrates a vinylogous effect previously observed only in small molecules, as supported by DFT calculations and model compounds. This study is the first to report such a long‐range vinylogous effect for a polymer through the collective synergy of numerous remotely located functional groups. The extended π‐conjugation across the polymer backbone facilitates efficient charge transport, enabling PIDHPTT to achieve balanced ambipolar OFET mobilities (μ_h_=0.021 cm^2^V^−1^s^−1^ and μ_e_=0.029 cm^2^V^−1^s^−1^).

PIDHPTT′s favorable HOMO energy level and high hydrophobic side chains render the polymer highly stable in aqueous environments, making it an ideal organic semiconductor material for p‐type OFET‐based aqueous sensors. DFT simulations revealed the strong binding preference of PIDHPTT for fluoride ions over chloride and bromide ions, highlighting the importance of the conjugation transformation from lactam to lactim for achieving high fluoride sensitivity. Experimental data demonstrated that PIDHPTT exhibits significant changes in optical absorption, electrochemical impedance, and charge transport in response to fluoride ions, distinguishing these responses from those with other halide ions. WG‐OFET sensors exhibited a LOD for fluoride ions as low as 0.28 μM, which is significantly lower than the WHO recommended fluoride level in drinking water.

Overall, this study demonstrates the potential of using a non‐conjugated lactam building block to construct a fully conjugated polymer semiconductor via the collective vinylogous effects of lactim DHP units through π‐bridges, particularly for detecting fluoride ions in aqueous solutions. The findings offer a promising approach to developing a new class of fully conjugated polymer semiconductors by employing non‐conjugated lactam building blocks through long‐range self‐tautomerization, potentially opening new application opportunities for polymer semiconductors.

## Supporting Information

The data that support the findings of this study are available in the supplementary material of this article.

Details on characterization methods, materials synthesis, device fabrication, and additional data on UV/Vis, NMR, OFET and WG‐OFET characteristics, DFT calculations, and EIS measurements.

## Conflict of Interests

The authors declare no conflict of interest.

1

## Supporting information

As a service to our authors and readers, this journal provides supporting information supplied by the authors. Such materials are peer reviewed and may be re‐organized for online delivery, but are not copy‐edited or typeset. Technical support issues arising from supporting information (other than missing files) should be addressed to the authors.

Supporting Information

## Data Availability

The data that support the findings of this study are available in the supplementary material of this article.
