# Supplementary material for: Full Conjugation in a Polymer with Non‐conjugated Piperazine‐2,5‐dione Units via Energy‐minimized Lactam‐to‐Lactim Tautomerization Enables Water‐gated Transistor Fluoride Sensors
Source: Angew Chem Int Ed Engl. 2024 Dec 5;64(7):e202419314. doi: 10.1002/anie.202419314 (PMC11811691; doi:10.1002/anie.202419314)
Supplement: Supplementary file 1 — Supporting Information [file ANIE-64-e202419314-s001.pdf]

Angewandte Chemie
Eine Zeitschrift der Gesellschaft Deutscher Chemiker

Supporting Information

# Full Conjugation in a Polymer with Non-conjugated Piperazine-2,5-dione Units via Energy-minimized Lactam-to-Lactim Tautomerization Enables Water-gated Transistor Fluoride Sensors

*N. Zhao, S. J. Jeon, Y. Yuan, S. Venkateswarlu, A. Stella, J. Papazotos, Y. Li**

Copyright WILEY-VCH Verlag GmbH & Co. KGaA, 69469 Weinheim, Germany, 2018.

Supporting Information

# Full Conjugation in a Polymer with Non-conjugated Piperazine-2,5-dione Units via Energy-minimized Lactam-to-Lactim Tautomerization Enables Water-gated Transistor Fluoride Sensors

Naixin Zhao, Sung Jae Jeon, Yi Yuan, Samala Venkateswarlu, Andrew Stella, Jimmy Papazotos, and Yuning Li*

N. Zhao, Dr. S. J. Jeon, Y. Yuan, Dr. S. Venkateswarlu, A. Stella, J. Papazotos, Prof. Y. Li
Department of Chemical Engineering
Waterloo Institute for Nanotechnology (WIN)
University of Waterloo
200 University Ave West, Waterloo, Ontario N2L 3G1, Canada
E-mail: yuning.li@uwaterloo.ca
*Corresponding author

## 1. Materials

Glycine anhydride (GA) (or piperazine-2,5-dione), 6-bromoisatin, 11-(bromomethyl)tricosane, 2,5-bis(trimethylstannyl)-thieno[3,2-b]thiophene were purchased from commercial sources (Aldrich, TCI, VWR, and Oakwood) and used without further purification, unless stated otherwise. Anhydrous solvents were purchased from Sigma-Aldrich and used as received. 1,4-Diacetylpiperazine-2,5-dione was synthesized according to the previously reported procedure.[1] A heavily n-doped Si wafer (4-inch) with a 300 nm polished $SiO_2$ layer was purchased from University Wafer. Chromium rods and gold ingots (99.9%) used for thermal evaporation was purchased from Angstrom Engineering.

## 2. Characterizations

### 2.1 Instruments

Nuclear magnetic resonance (NMR) spectra were obtained using a Bruker DPX 300 MHz spectrometer. UV-Vis spectra were obtained using a Cary 7000 UMS UV-Vis-NIR spectrophotometer. Cyclic voltammetry (CV) measurements were performed using an indium-doped tin oxide (ITO)-coated glass working electrode, an Ag/AgCl reference electrode, and a Pt disk counter electrode in a 0.1 M tetrabutylammonium hexafluorophosphate solution in anhydrous acetonitrile at a scan rate of 20 mV/s. Ferrocene was used as the reference, which has a HOMO energy level of -4.8 eV.[2] The HOMO energy level of the polymer was calculated using the equation of $E_{HOMO}$ (eV) = -($E_{oxi.onset}$ – $E_{Fc/Fc+onset}$) – 4.8 eV, where $E_{Fc/Fc+onset}$ was determined by scanning at same scan rate with blank electrodes plus 50 mg of ferrocene added to the electrolyte (**Figure S6**). The resulting CV diagram has an oxidative onset voltage of -0.02 V corresponding to a $E_{Fc/Fc+onset}$ = -0.02 eV. Matrix-assisted laser desorption ionization time-of-flight mass spectrometry (MALDI-TOF MS) was performed using a Bruker Autoflex Speed MALDI-TOF mass spectrometer using dithranol dissolved in tetrahydrofuran as the matrix. Two-dimensional grazing incidence X-ray diffraction (2D-GIXD) measurements were performed on a Bruker D8 Advance diffractometer with Cu Ka radiation ($\lambda$ = 0.15406 nm) using polymer films spin coated on a silicon dioxide wafer. Atomic force microscopy (AFM) images were taken with a Dimension 3100 scanning probe microscope. Transistor measurements were performed using an Agilent B2912A Semiconductor Analyzer. The molecular weight of the polymer was measured using a high-temperature gel permeation chromatography (HT-GPC) system on an Agilent PL-GPC 220 at 150 °C using 1,2,4-trichlorobenzene (TCB) as eluent. Thermogravimetric analysis (TGA) was performed on a TGA Q500 (TA Instruments) in nitrogen at a heating rate of 10 °C/min.

Computer simulations were carried out by Gaussian 09 software using the density functional theory (DFT), as approximated by the B3LYP functional, and using the 6-31G (d) basis set. The binding energies were calculated by firstly using Merck Molecular Force Field 94 (MMFF94) molecular mechanic function via Avogadro (version 1.2.0) to obtain an optimized molecular geometry between the target molecule and halide ion. Then, the geometries were further optimized with Gaussian 09 using the same method described previously.

### 2.2 Fabrication of organic field effect transistors

Gold source and drain contacts were patterned on a heavily n-doped Si/$SiO_2$ wafer, with the doped Si layer serving as the gate electrode and a 300 nm-thick $SiO_2$ layer as the gate dielectric, using a conventional photolithography process. Then, metals were deposited on the pattern through thermal evaporation by first depositing chromium and then gold. The patterned devices were cleaned by submerging and sonicating them in deionized water for 20 minutes. The process was repeated with acetone and isopropanol. The cleaned substrates were then dried with compressed nitrogen and treated with oxygen plasma under low air flow for 2 minutes. Then, substrates were immersed in 100 mL deionized water covered by a petri dish, and 2~3 drops of 1: 10: 10 ($HNO_3$: HCl: $H_2O$) acid solution was added. Acid treatment was allowed for roughly 2~3 minutes before the residue acid was rinsed off with deionized water and then isopropanol. The substrates were dried with compressed nitrogen and heated on a hot plate at 120 °C for 15 minutes. Finally, the substrate surface was modified with a self-assembled monolayer (SAM) by immerging the substrate in a trichlorododecylsilane (DDTS) solution in toluene (~10 mM) for 25 minutes. Residue chemicals were washed off with toluene and the transistor substrate was ready to use.

PIDHPTT thin films were deposited onto the substrates by spin-coated (3000 rpm) 50 µL of polymer solution (5 mg/mL in chloroform) for 100 seconds, yielding a thin film with a thickness of approximately 30-50 nm. The thermal annealing process was carried out by heating the substrate with the polymer film on a hotplate placed in a nitrogen-filled glove box for 20 minutes at different temperatures (50, 100, 150, 200, and 250 °C). Transistor performance was measured in the same glove box. The hole and electron mobilities were calculated in the saturation regime according to the following equation:

$$I_{DS} = \mu \frac{WC_i}{2L}(V_G - V_T)^2$$

where $I_{DS}$ is the drain-source current, µ is the charge carrier mobility, $C_i$ is the gate dielectric layer capacitance per unit area (~11.6 $nFcm^{-2}$ for DDTS modified $SiO_2$), $V_G$ is the gate voltage, $V_T$ is the threshold voltage, $L$ is the channel length (30 µm), and $W$ is the channel width (1000 µm).

The water-gated organic field effect transistor (WG-OFET) sensor devices were fabricated similarly, except that bare $SiO_2$ was used (without DDTS modification). Additionally, an interdigitated source/drain electrode pattern was employed with a channel length of 30 µm and a channel width of 15.8 mm. A 20 µL droplet of Milli-Q water was deposited as the gate dielectric, and the probe tip was positioned on top of the water droplet as the gate electrode. For the sensing experiment, 5 µL of analyte aqueous solution was added to the water droplet using a micropipette.

## 3. Synthesis

*3.1 Synthesis of 6-bromo-1-(2-decyltetradecyl)indoline-2,3-dione*

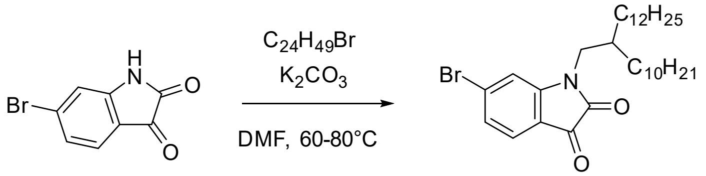


To a two-necked 250 mL round-bottom flask, 6-bromoisatin (1.5 g, 6.64 mmol) and potassium carbonate (2.3 g, 16.6 mmol) were added. The system was purged with $N_2$ before anhydrous DMF

(20 mL) was syringe injected through the rubber septum to suspend the mixture. The suspension was heated with stirring at 60 °C for 30 min until all the solids were dissolved. Then, 11-(bromomethyl)tricosane (3.6 g, 8.63 mmol) was added dropwise through a rubber stopper. The mixture was then heated under stirring at 80 °C for 12 hr. The reaction mixture was allowed to cool down to room temperature before being quenched with 1M HCl solution. The product was extracted with dichloromethane (DCM) and the organic layer was washed with deionized water twice and dried over anhydrous $Na_2SO_4$. The solution was filtered, and DCM was removed by rotary evaporation. The crude product was then purified with column chromatography on silica gel using 30% DCM in hexane as an eluent to afford the title compound (2.5 g, 68%) as orange oil. $^1$H NMR (300 MHz, Chloroform-*d*): δ = 7.47-7.45 (d, 1H), 7.28 (d, 1H), 7.03 (d, 1H), 3.59-3.56 (d, 2H), 1.84 (s, 1H), 1.40-1.17 (m, 40H), 0.93-0.84 (t, 6H)

*3.2 Synthesis of 3,6-bis((Z)-6-bromo-1-(2-decyltetradecyl)-2-oxoindolin-3-ylidene)piperazine-2,5-dione (**IDHP-Br**)*

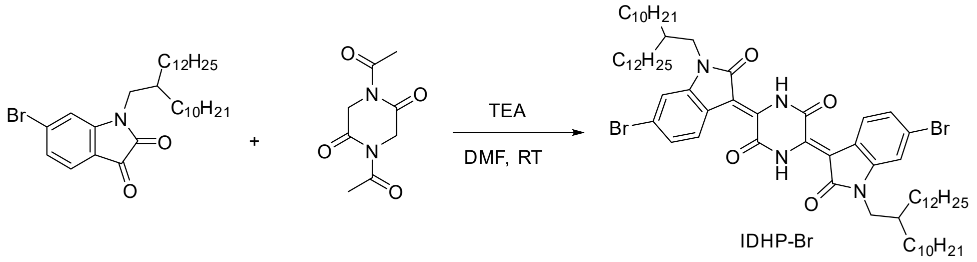


To a two-necked 100 mL round-bottom flask, 1,4-diacetylpiperazine-2,5-dione (0.18 g, 0.89 mmol) and 6-bromo-1-(2-decyltetradecyl)indoline-2,3-dione (1.0 g, 1.78 mmol) were added. The system was purged with $N_2$ before anhydrous DMF (15 mL) was added to dissolve the mixture, yielding a clear orange solution. Then, triethylamine (0.52 mL, 3.55 mmol) was added dropwise under stirring. The transparent orange solution quickly turned to a dark red color with precipitation observed during the addition. The reaction was allowed to proceed for 12 hours at room temperature before being quenched by precipitating in acetone. The crude product was obtained by filtration under reduced pressure and purified by column chromatography on silica gel using chloroform as eluent to afford IDHP-Br (1.0 g, 94%) as a red solid. $^1$H NMR (300 MHz, Chloroform-*d*): δ = 13.37 (s, 2H), 8.81-8.78 (d, 2H), 7.23 (d, 2H), 6.99 (d, 2H), 3.67-3.65 (d, 4H), 1.89 (s, 2H), 1.37-1.16 (m, 80H), 0.89-0.84 (t, 12H). Matrix-assisted laser desorption ionization mass spectrometry (MALDI-MS) was used to characterize the mass of IDHP-Br: Calculated [$M^+$]: 1203.45; Found: 1203.81.

*3.3 Synthesis of PIDHPTT*

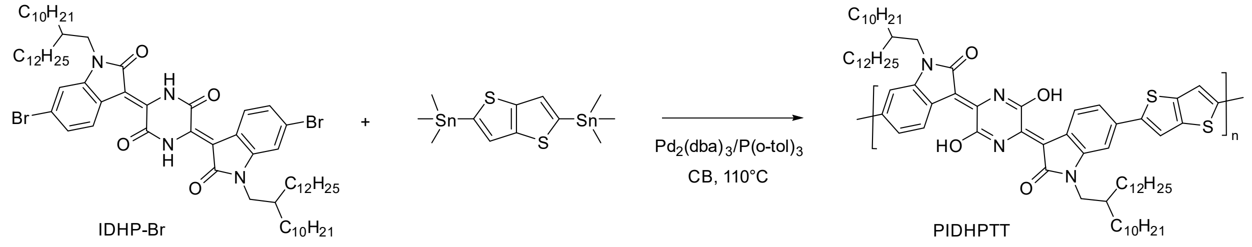


To a two-necked 25mL round-bottom flask, IDHP-Br (180.5 mg, 0.15 mmol), 2,5-bis(trimethylstannyl)-thieno[3,2-b]thiophene (69.9 mg, 0.15 mmol), and tri(o-tolyl)phosphine (P(o-tol)$_3$) (3.7 mg, 0.012 mmol) were added. The system was purged with argon before anhydrous

chlorobenzene (CB) (4 mL) was syringe injected through the rubber septum to dissolve the mixture. In an argon-filled glove box, tris(dibenzylideneacetone)dipalladium(0) ($Pd_2dba_3$) (5.5 mg, 0.006 mmol) was pre-dissolved in 2 mL anhydrous CB and was syringe injected into the reaction mixture. The mixture was then heated at 110 °C with stirring for 48 hours. Then, the mixture was allowed to cool down to room temperature before precipitating from methanol. The crude polymer product was collected by filtration, and purified with Soxhlet extraction using acetone, hexane, and chloroform successively. The chloroform portion was collected, yielding the purified PIDHPTT as dark green flakes (151 mg, 85%).

*3.4 General synthesis procedure of IDHP-2T and IDHPTT-dimer, and IDHPTT-tetramer*

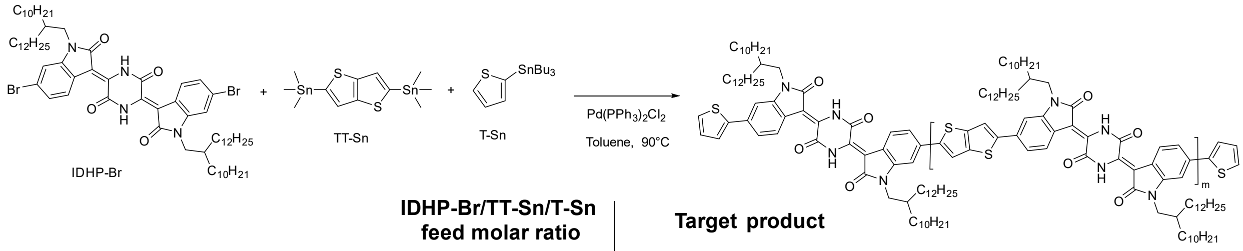


| IDHP-Br/TT-Sn/T-Sn feed molar ratio | Target product |
|---|---|
| 1/0/2 | IDHP-2T: m = 0 |
| 2/1/2 | IDHPTT-dimer: m = 1 |
| 4/3/2 | IDHPTT-tetramer: m = 3 |

**Scheme S1**. Synthetic routes to IDHP-2T and IDHPTT-dimer, and IDHPTT-tetramer.

To a two-necked 25mL round-bottom flask, IDHP-Br (50 mg, 0.042 mmol), 2,5-bis(trimethylstannyl)-thieno[3,2-b]thiophene (TT-Sn) (0, 0.5, or 0.75 molar eq.), and 2-(tributylstannyl)thiophene (T-Sn) (2, 1, or 0.5 molar eq.) were added. The system was purged with argon before anhydrous toluene (4 mL) was syringe injected through the rubber septum to dissolve the mixture. In an argon-filled glove box, Bis(triphenylphosphine)palladium(II) dichloride ($Pd(PPh_3)_2Cl_2$) (0.03 molar eq.) was pre-dissolved in 1 mL anhydrous toluene and was syringe injected into the reaction mixture. The mixture was then heated at 90 °C with stirring for 24 hours. Upon completion, the mixture was allowed to cool down to room temperature before extracting with chloroform. The organic layer was washed with 1M hydrochloric acid solution and dried with sodium sulphate. Residue chloroform was removed under reduced pressure and the crude product was purified by column chromatography on silica gel using toluene as eluent to afford the target product, IDHP-2T (m = 0 with IDHP-Br : TT-Sn : T-Sn = 1 : 0 : 2) (estimated 45% yield), IDHPTT-dimer (m = 1 with IDHP-Br : TT-Sn : T-Sn = 2 : 1 : 2;) (estimated 48% yield), or IDHPTT-tetramer (m = 3 with IDHP-Br : TT-Sn : T-Sn = 4 : 3 : 2) (estimated 59% yield).

## 4. Additional data

**Table S1.** Summary of UV-Vis optical and CV electrochemical properties of IDHPTT monomeric, oligomeric species, and PIDHPTT.

| | $E_{g,opt}$ (eV) | $\lambda_{max}$ (nm) | $\lambda_{onset}$ (nm) | $HOMO_{CV}$ (eV) | $LUMO_{CV}$ (eV) |
|---|---|---|---|---|---|
| IDHP-Br | 1.99 | 417 | 622 | n.a. | n.a. |
| IDHP-2T | 1.81 | 537 | 686 | n.a. | n.a. |
| IDHPTT-dimer | 1.62 | 625 | 767 | n.a. | n.a. |
| IDHPTT-tetramer | 1.60 | 633 | 775 | n.a. | n.a. |
| PIDHPTT | 1.57 | 718 | 789 | -5.58 | -3.71 |

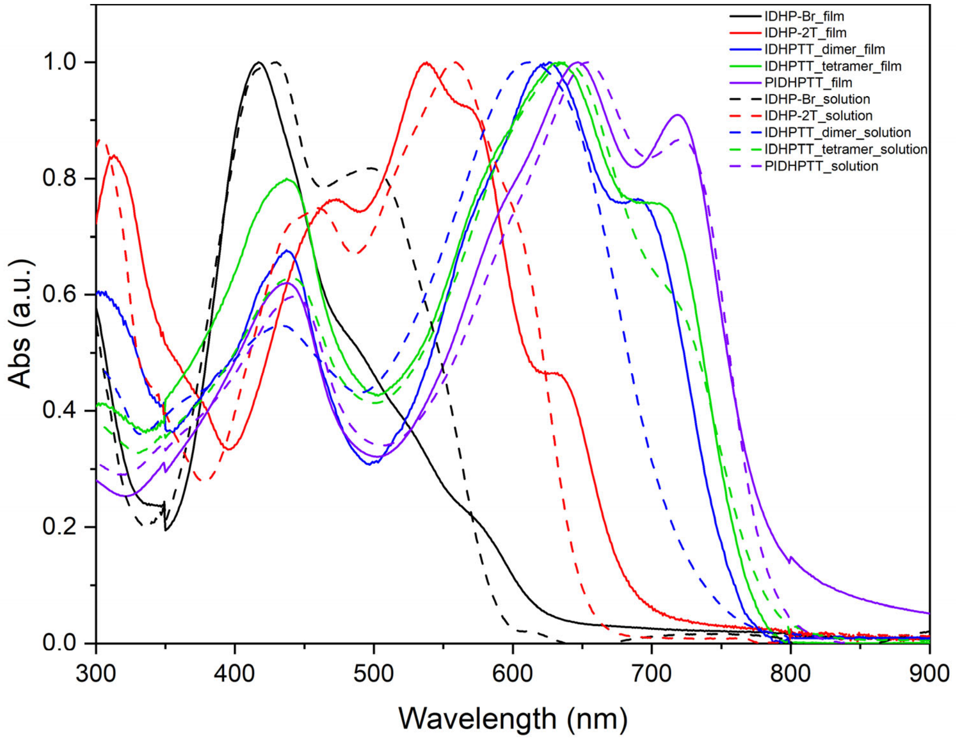


**Figure S1.** UV-Vis absorption spectra of IDHP-derived compounds in solution (chloroform) and in the thin-film state. Data are summarized in Table S1. The redshift in the spectra and the decreasing bandgaps with increasing backbone length indicate enhanced π-conjugation.

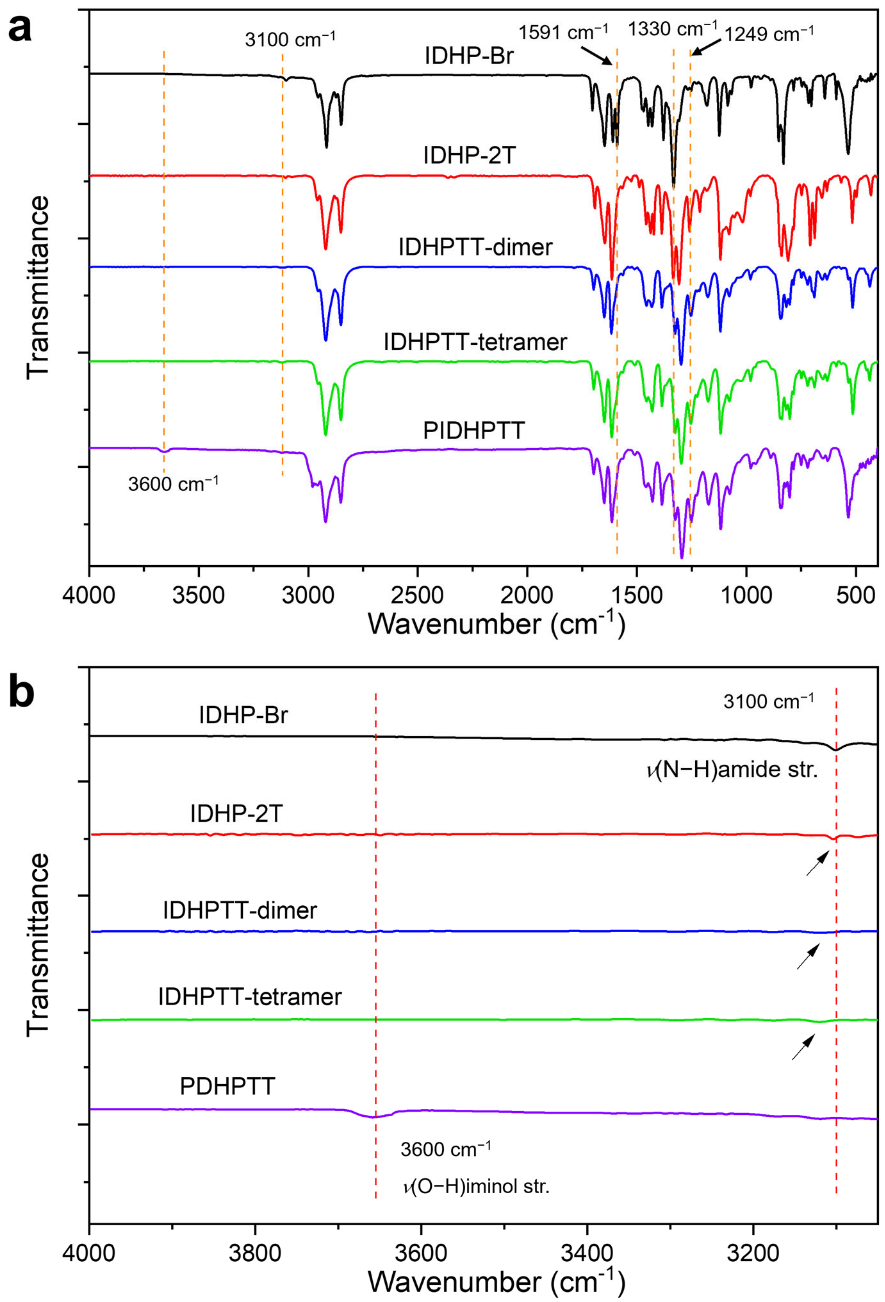


**Figure S2.** FTIR spectra of IDHP-derived compounds in the ranges of (a) 450 to 4000 $cm^{-1}$ and (b) 3150 to 4000 $cm^{-1}$. Major peak assignments: 3600 $cm^{-1}$: ν(O−H)iminol stretching;[3,4] 3100 $cm^{-1}$: ν(N−H)amide stretching;[4,5] 1591 $cm^{-1}$: ν(N−H)amide bending;[4,5] 1330 $cm^{-1}$: ν(C−N)amide bending;[4,5] 1249 $cm^{-1}$: ν(C−O(H))iminol bending.[6,7] The decreasing intensities of the N−H and C−N peaks, coupled with the increasing intensities of the O−H and C−O(H) peaks, indicate a gradual conversion from the lactam form to the lactim form as the backbone length increases.

**Table S2.** Summary of DFT simulated energies for oligomers for PIDHPTT from monomer to tetramer in two tautomer forms.

Lactam

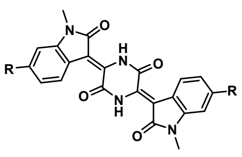

IDHP-H-NH: R = H
IDHP-Br-NH: R = Br

Lactim

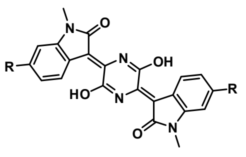

IDHP-H-OH: R = H
IDHP-Br-OH: R = Br

Lactam

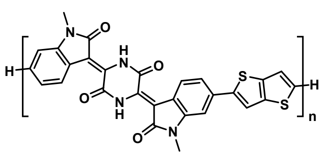

(IDHPTT-NH)$_n$: n = 1, 2, 3, or 4

Lactim

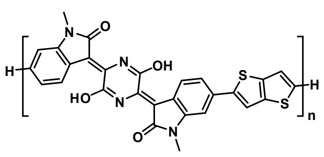

(IDHPTT-OH)$_n$: n = 1, 2, 3, or 4

| Species | Tautomer | DFT Energy (Hartree) | Total Energy Difference (kJ mol$^{-1}$) | Energy Difference per Unit (kJ mol$^{-1}$) | HOMO (eV) | LUMO (eV) | Band Gap (eV) | C-N Bond Length (Å) | C-O Bond Length (Å) |
|---|---|---|---|---|---|---|---|---|---|
| IDHP-H | IDHP-H-NH | -1367.9644 | 186.1480 | 186.1480 | -5.76024 | -3.04849 | 2.6918 | 1.37985 ± 0 | 1.22334 ± 0 |
| | IDHP-H-OH | -1367.8935 | | | -5.27792 | -3.17965 | 2.0983 | 1.29033 ± 0 | 1.34746 ± 0 |
| IDHP-Br | IDHP-Br-NH | -6510.1739 | 184.0476 | 184.0476 | -5.91630 | -3.30564 | 2.6107 | 1.38004 ± 0 | 1.22320 ± 0 |
| | IDHP-Br-OH | -6510.1038 | | | -5.47765 | -3.43489 | 2.0428 | 1.29077 ± 0 | 1.34716 ± 0 |
| Monomer | IDHPTT-NH | -2394.1787 | 184.5727 | 184.5727 | -5.41452 | -3.08931 | 2.3252 | 1.38002 ± 0.00033 | 1.22369 ± 0.00035 |
| | IDHPTT-OH | -2394.1084 | | | -5.12608 | -3.22428 | 1.9018 | 1.29141 ± 0.00028 | 1.34808 ± 0.00055 |
| Dimer | (IDHPTT-NH)$_2$ | -4787.1698 | 367.0449 | 183.5225 | -5.24228 | -3.19734 | 2.0449 | 1.38016 ± 0.00054 | 1.22384 ± 0.00034 |
| | (IDHPTT-OH)$_2$ | -4787.0300 | | | -4.97261 | -3.36578 | 1.6068 | 1.29212 ± 0.00072 | 1.34829 ± 0.00057 |
| Trimer | (IDHPTT-NH)$_3$ | -7180.1350 | 479.6789 | 159.8930 | -5.25779 | -3.27326 | 1.9845 | 1.38338 ± 0.00518 | 1.22437 ± 0.00658 |
| | (IDHPTT-OH)$_3$ | -7179.9523 | | | -4.93805 | -3.42782 | 1.5102 | 1.29328 ± 0.00172 | 1.34619 ± 0.00506 |
| Tetramer | (IDHPTT-NH)$_4$ | -9573.0564 | 435.3079 | 108.8270 | -5.29398 | -3.26591 | 2.0281 | 1.38983 ± 0.01029 | 1.21946 ± 0.00456 |
| | (IDHPTT-OH)$_4$ | -9572.8906 | | | -4.96744 | -3.5119 | 1.4555 | 1.29661 ± 0.00423 | 1.33957 ± 0.00979 |

**Table S3.** P-type and n-type transistor performance for PIDHPTT at different annealing temperatures (average values from five devices per data point).

| Mode | Annealing temp. (°C) | Max. $\mu_{sat}$ (cm$^2$ V$^{-1}$ s$^{-1}$) | Avg. $\mu_{sat}$ ± Std. (cm$^2$ V$^{-1}$ s$^{-1}$) | $V_T$ (V) | $I_{on/off}$ |
|---|---|---|---|---|---|
| P-type | RT | 0.0042 | 0.0032 ± 0.0008 | -55.4 | 1.00E+04 |
| | 50 | 0.0048 | 0.0035 ± 0.0008 | -54.4 | 10000 |
| | 100 | 0.0105 | 0.0070 ± 0.002 | -43.8 | 1000 |
| | 150 | 0.0139 | 0.0109 ± 0.0026 | -40.2 | 100 |
| | 200 | 0.0181 | 0.0150 ± 0.0026 | -34.3 | 100 |
| | 250 | 0.0245 | 0.0209 ± 0.0031 | -37.7 | 100 |
| N-type | RT | 0.0033 | 0.0027 ± 0.0007 | 37.7 | 1.00E+05 |
| | 50 | 0.0038 | 0.0027 ± 0.0006 | 39.3 | 10000 |
| | 100 | 0.0145 | 0.0080 ± 0.0037 | 39.3 | 1000 |
| | 150 | 0.0182 | 0.0138 ± 0.0038 | 38.9 | 1000 |
| | 200 | 0.0253 | 0.0203 ± 0.0043 | 35.2 | 100 |
| | 250 | 0.0349 | 0.0290 ± 0.0051 | 39.0 | 100 |

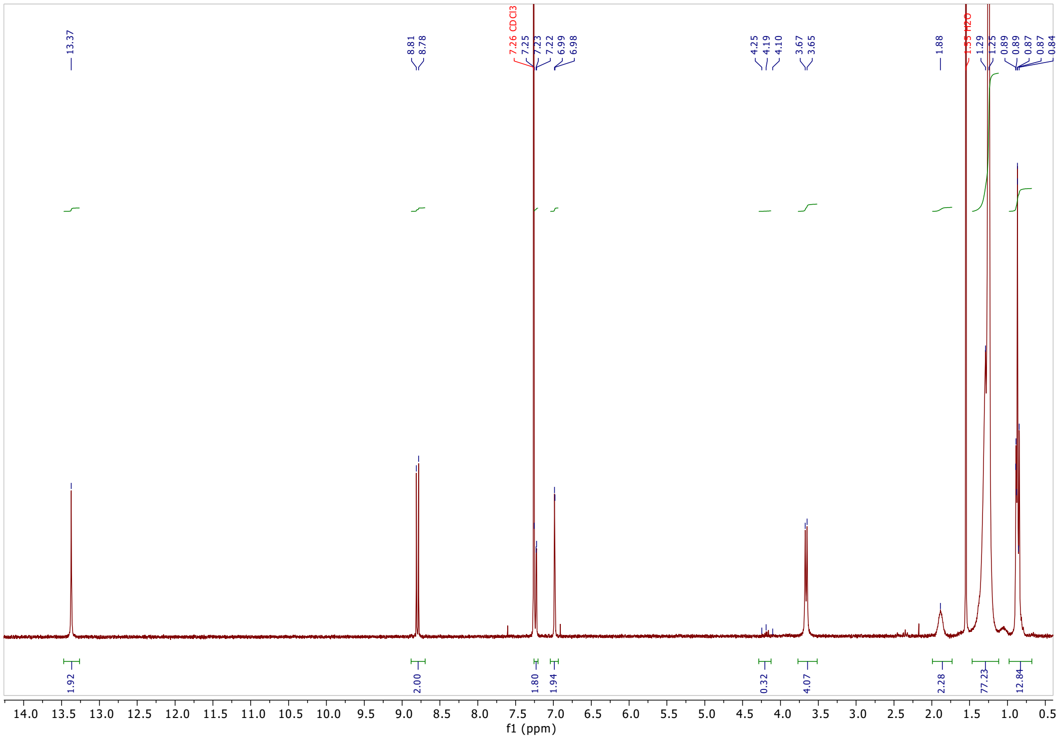

**Figure S3.** 300 MHz $^{1}H$ NMR spectrum of IDHP-Br monomer in chloroform-*d* at room temperature.

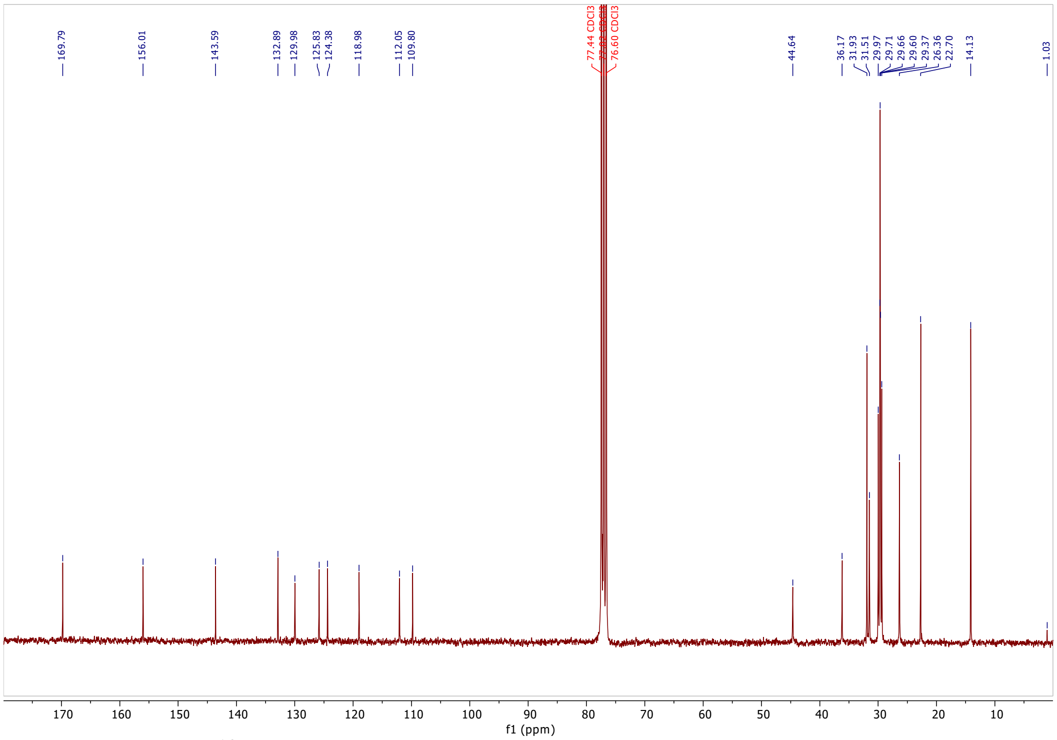

**Figure S4.** 300 MHz $^{13}C$ NMR spectrum of IDHP-Br monomer in chloroform-*d* at room temperature

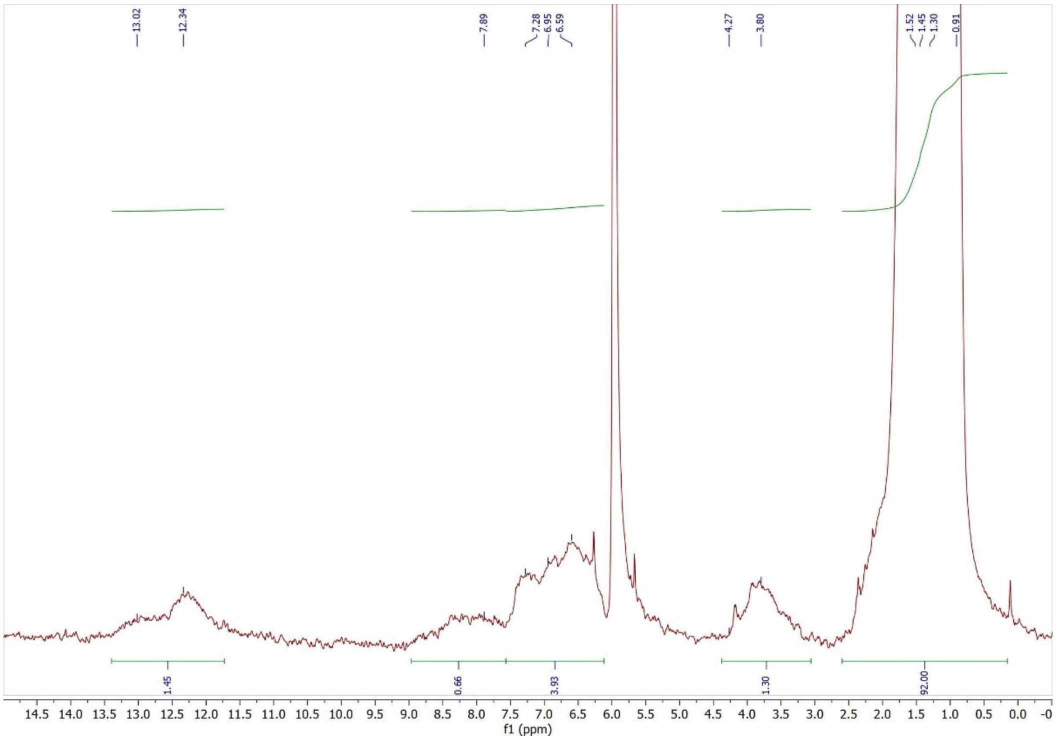


**Figure S5.** 300 MHz $^{1}H$ NMR spectrum of PIDHPTT in 1,1,2,2-tetrachloroethane-$d_2$ measured at 100 °C.

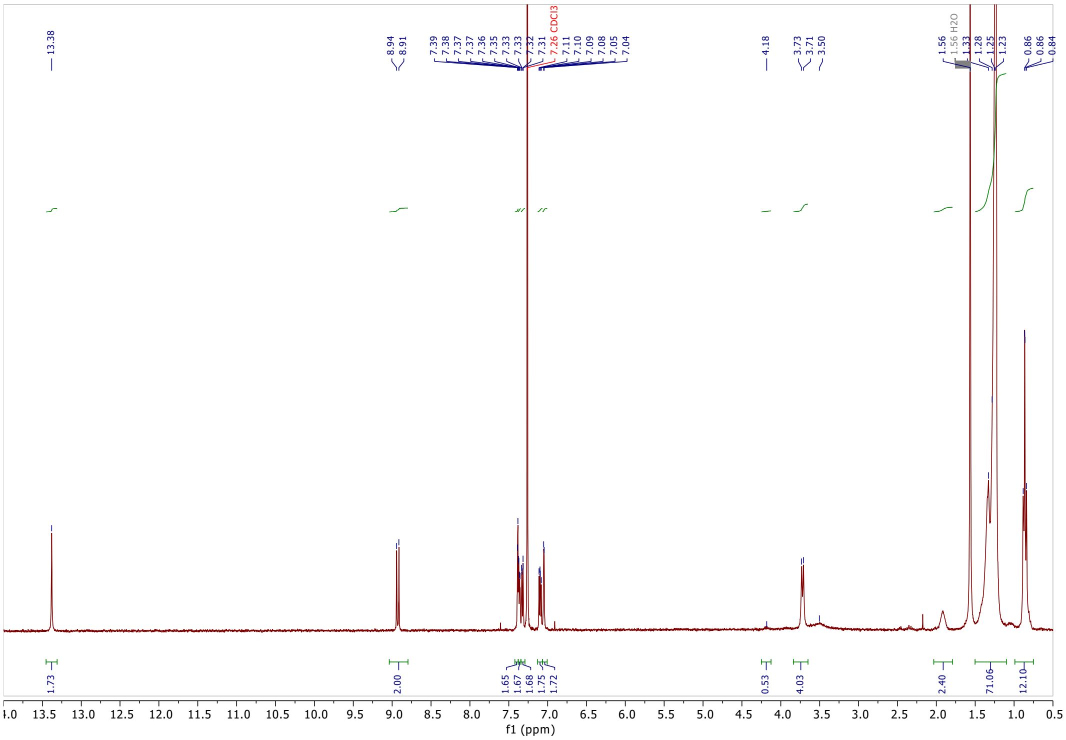


**Figure S6.** 300 MHz $^{1}H$ NMR spectrum of IDHP-2T monomer in chloroform-$d$ at room temperature.

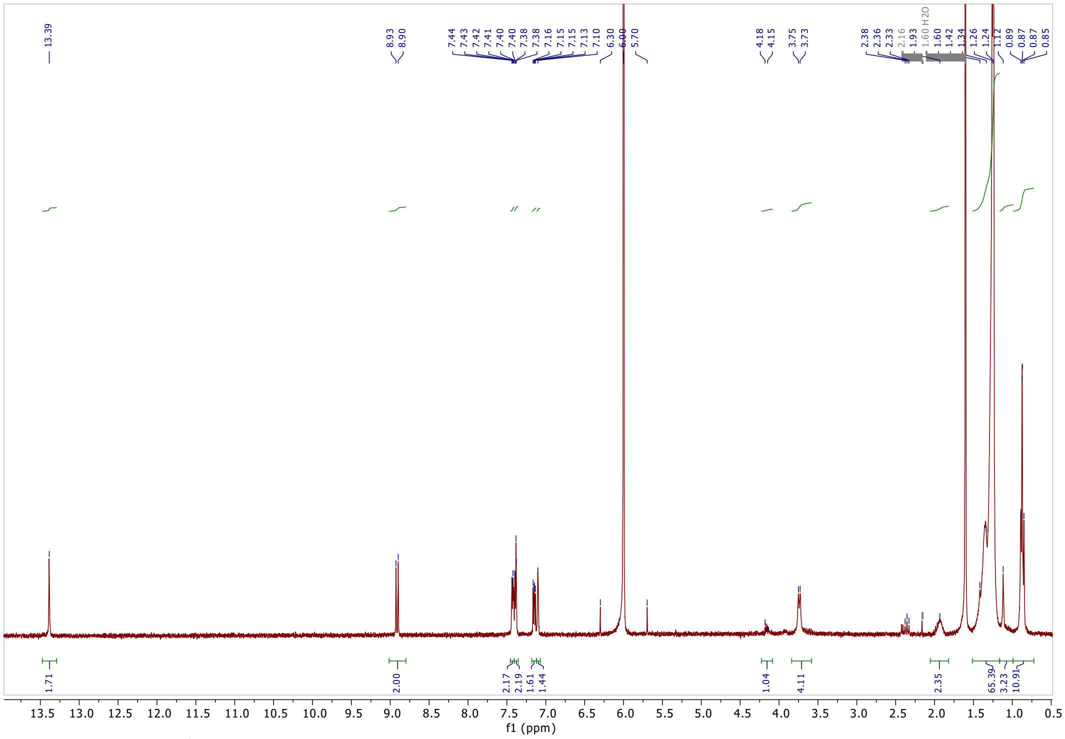


**Figure S7.** 300 MHz $^{1}H$ NMR spectrum of IDHP-2T monomer in 1,1,2,2-tetrachloroethane-$d_2$ at room temperature.

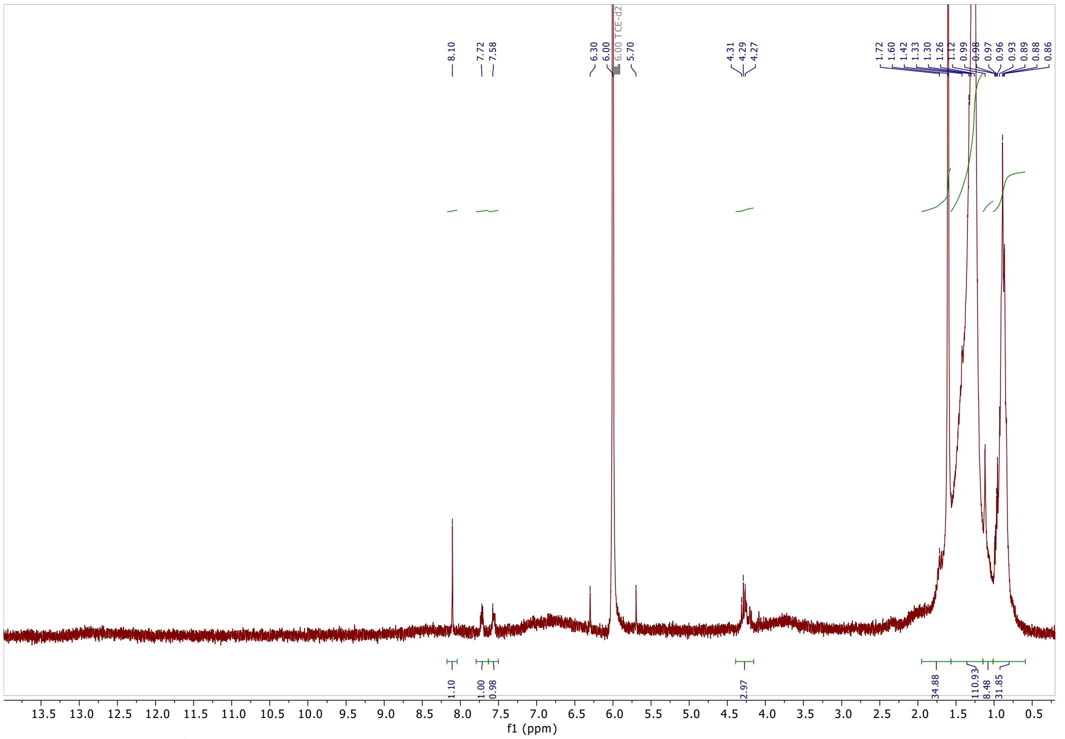


**Figure S8.** 300 MHz $^{1}H$ NMR spectrum of IDHPTT dimer in 1,1,2,2-tetrachloroethane-$d_2$ at room temperature.

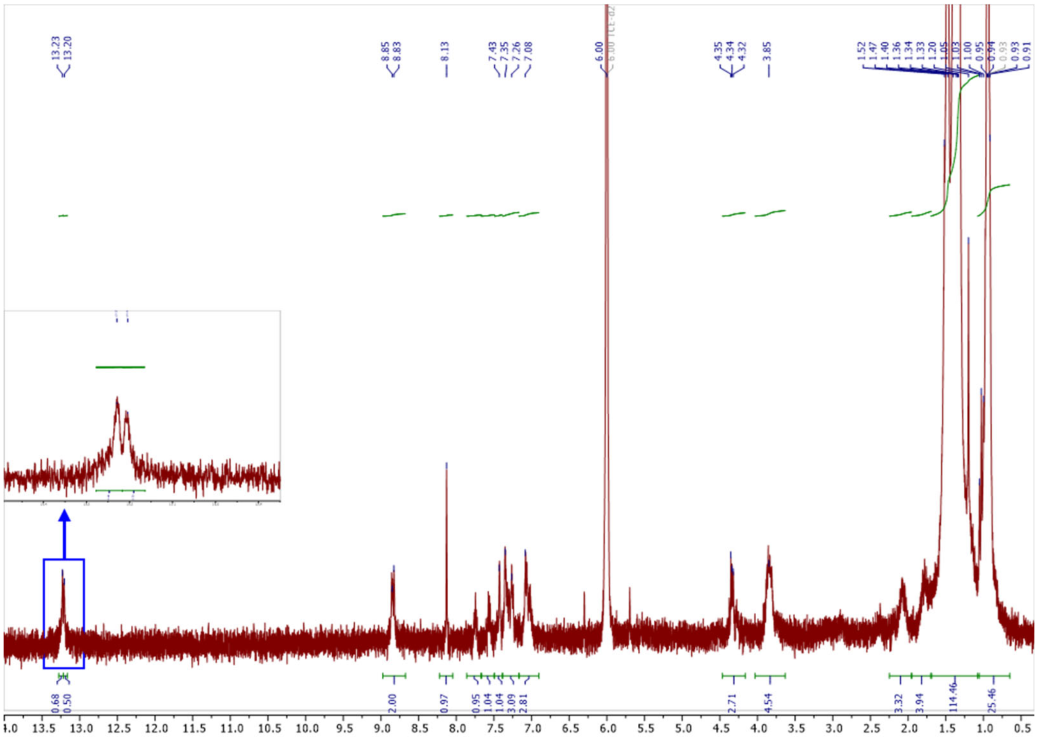

**Figure S9.** 300 MHz $^{1}$H NMR spectrum of IDHPTT dimer in 1,1,2,2-tetrachloroethane-$d_2$ measured at 100 °C.

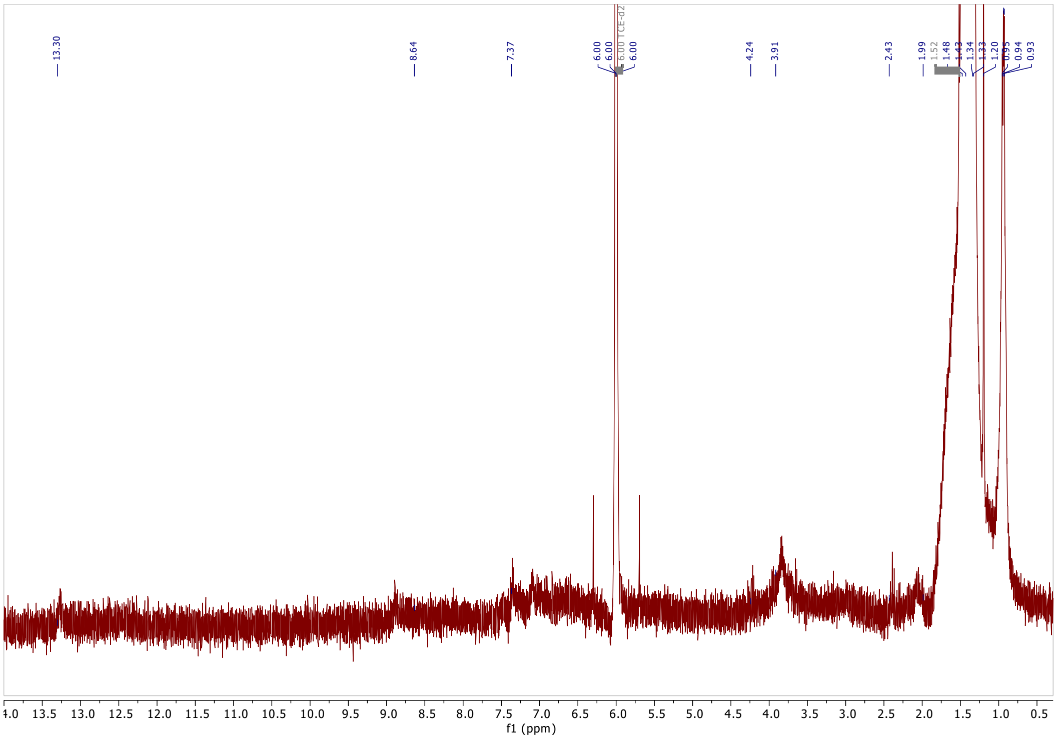


**Figure S10.** 300 MHz $^{1}$H NMR spectrum of IDHPTT tetramer in 1,1,2,2-tetrachloroethane-$d_2$ measured at 100 °C.

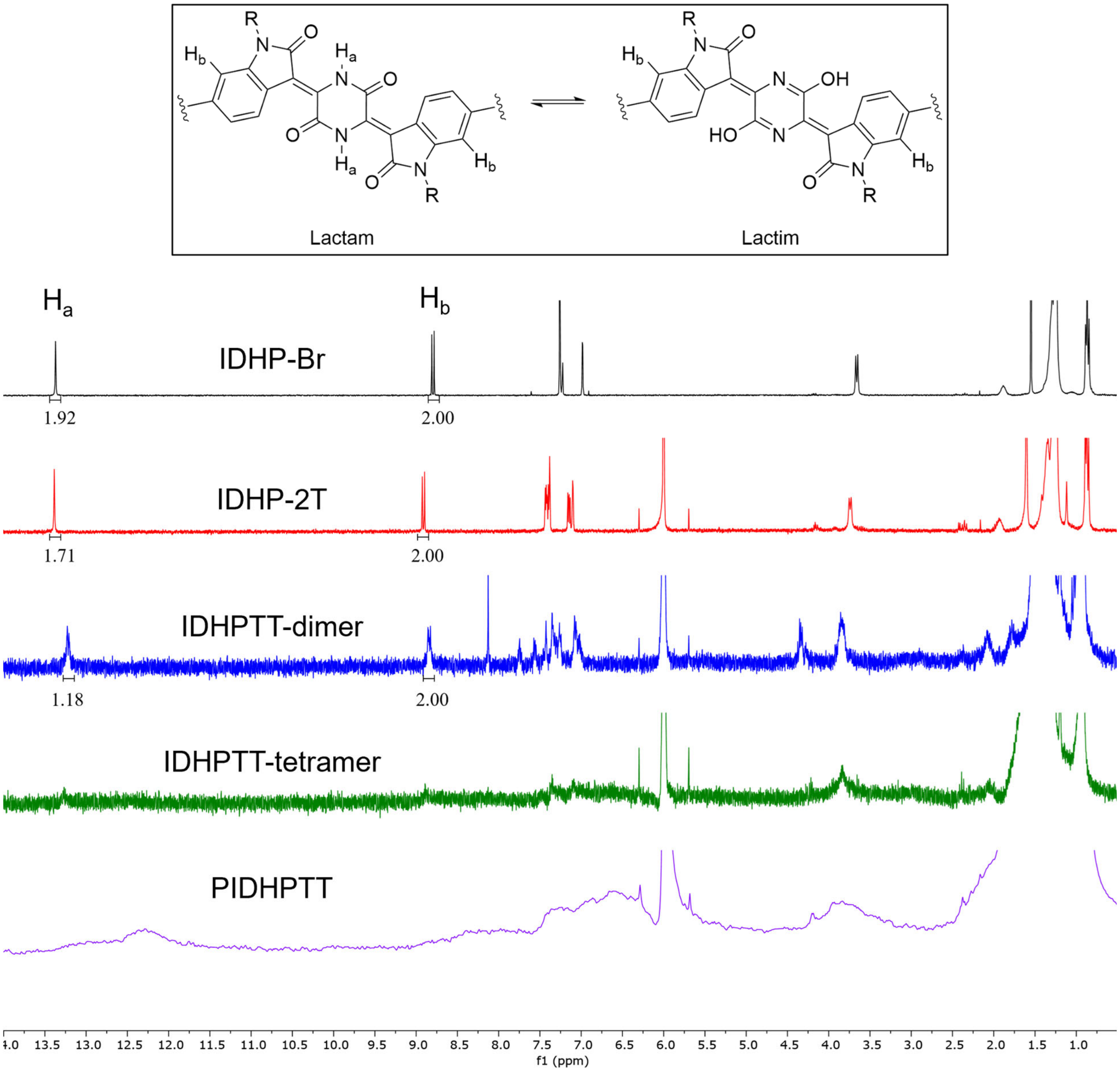


**Figure S11.** Compiled $^{1}$H NMR spectra of for IDHP-Br (Figure S3), IDHP-2T (Figure S7), IDHPTT-dimer (Figure S9), IDHPTT-tetramer (Figure S10), and PIDHPTT (Figure S5). Based on the ratio of $H_a$ and $H_b$ peak integrals, the lactam isomer content of the IDHP units is calculated to be 96% for IDHP-Br, 86% for IDHP-2T, and 59% for IDHP-dimer.

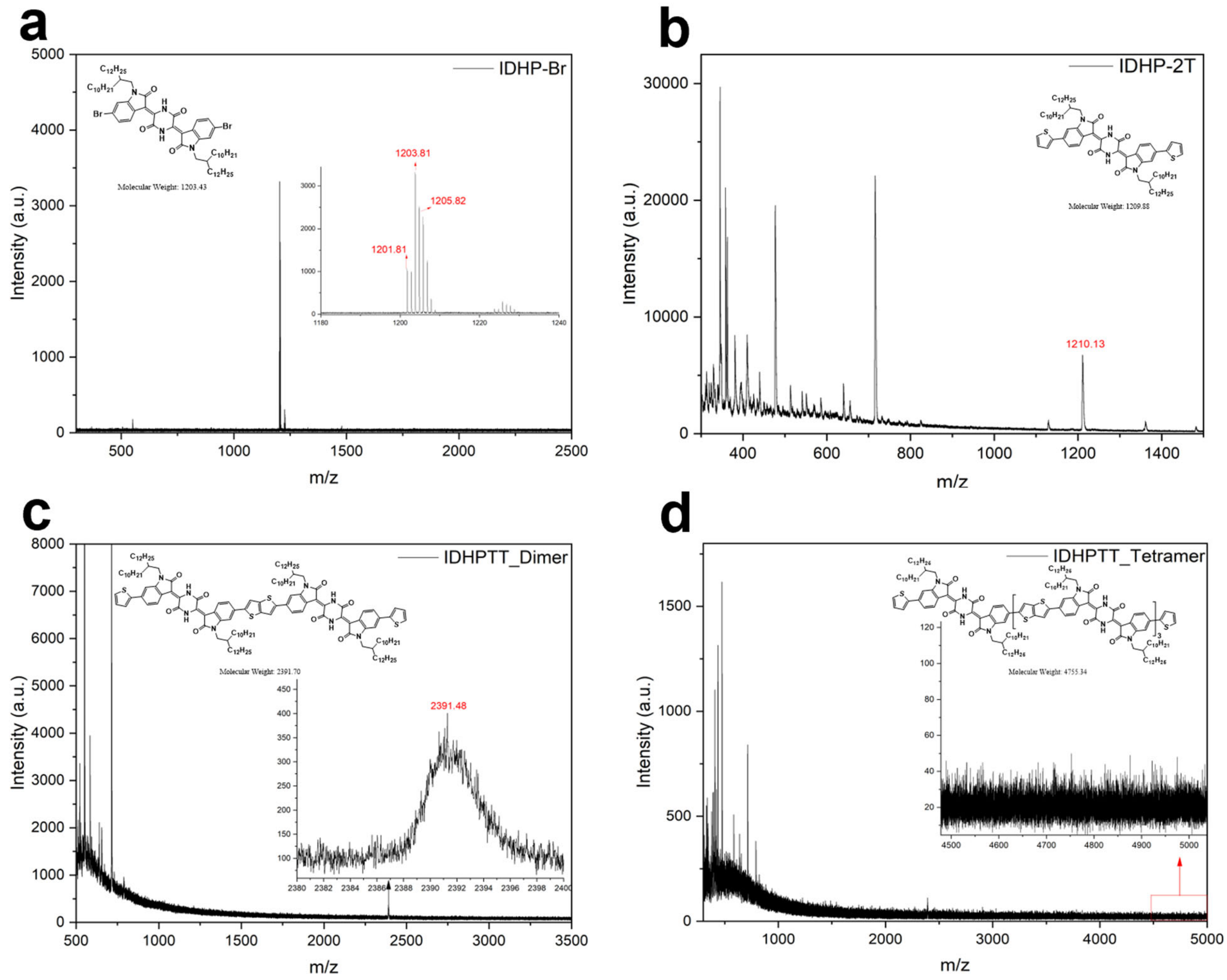


**Figure S12.** MALDI-TOF MS spectra of (a) IDHP-Br, (b) IDHP-2T, (c) IDHPTT-dimer, and (d) IDHPTT-tetramer.

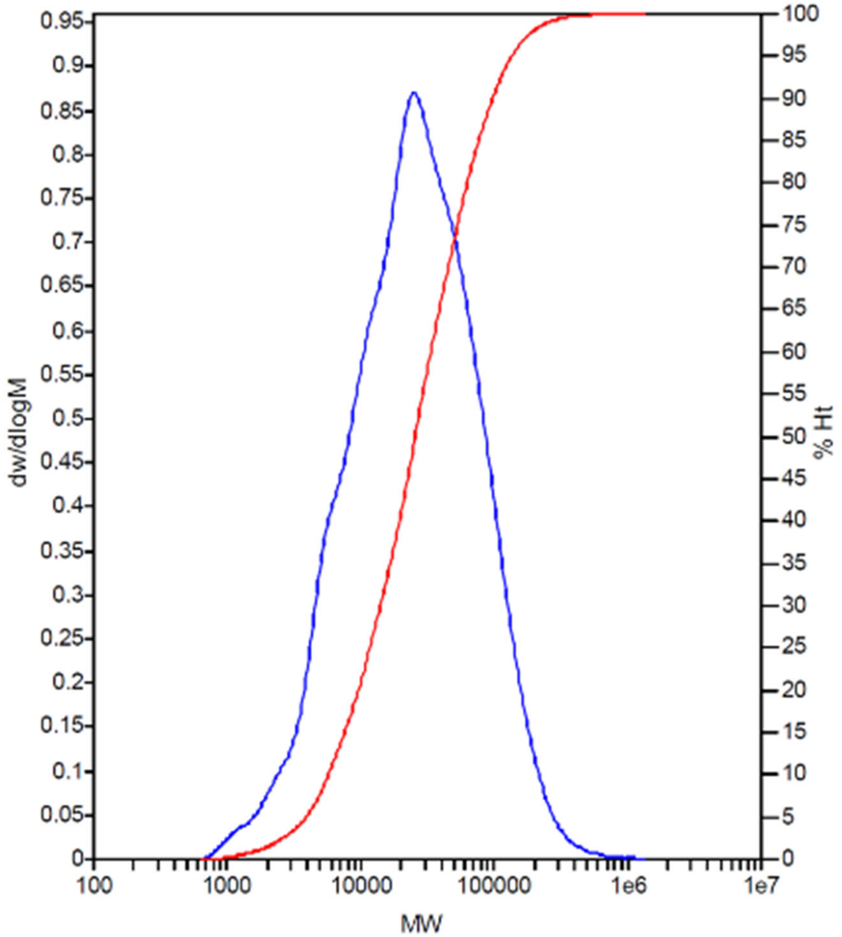


**Figure S13.** HT-GPC chromatogram of PIDHPTT measured at 150 °C using 1,2,4-trichlorobenzene (TCB) as eluent.

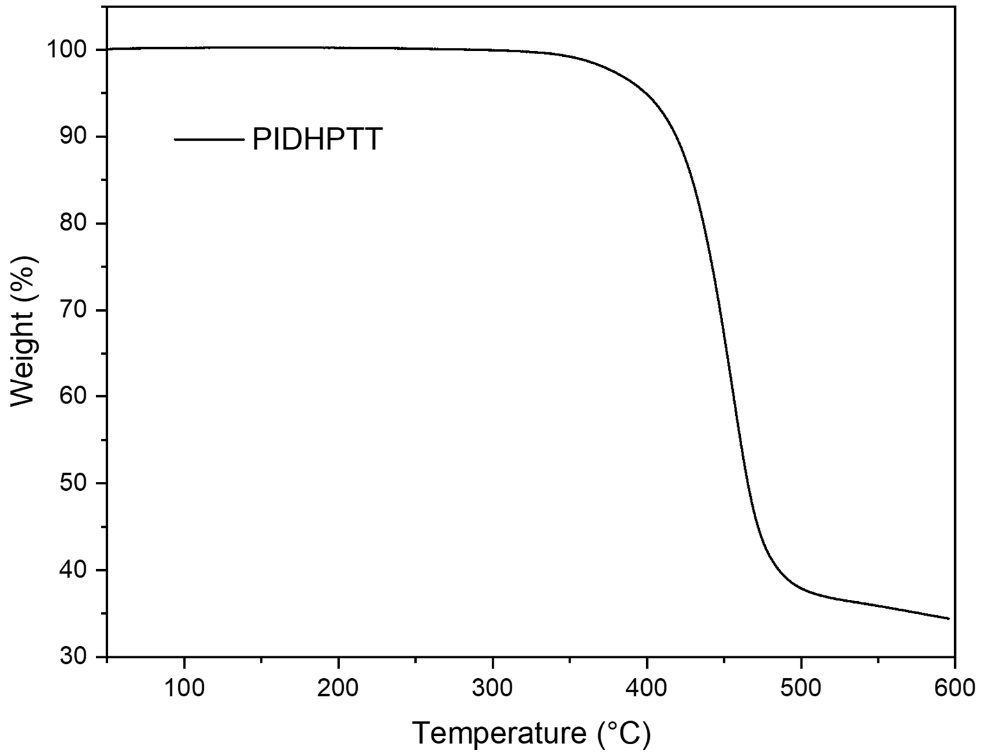


**Figure S14.** TGA curve of PIDHPTT measured in nitrogen at a heating rate of 10 °C min$^{-1}$.

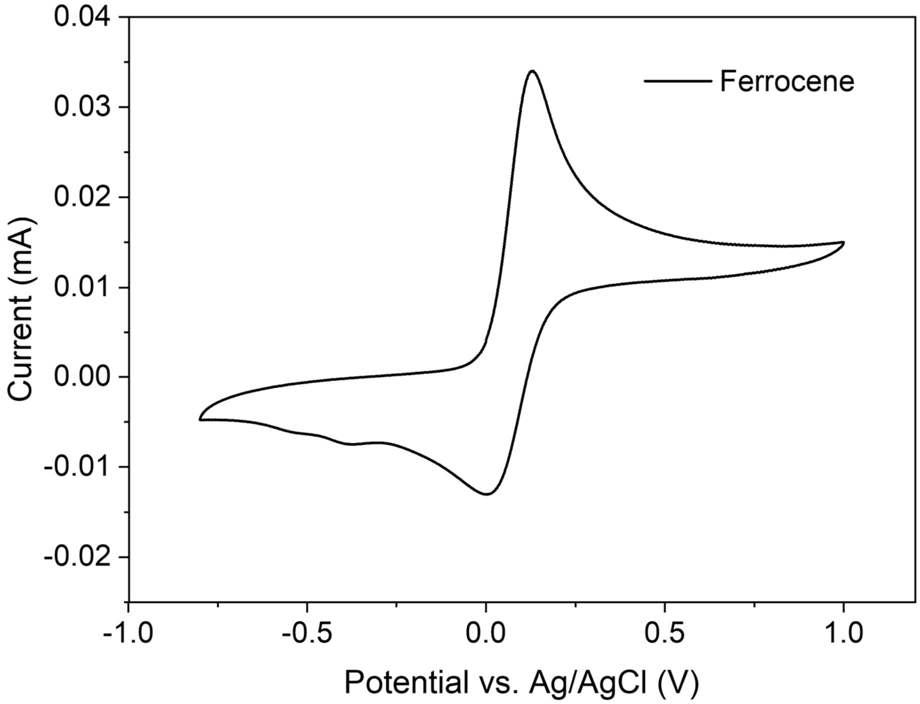


**Figure S15.** Cyclic voltammogram of ferrocene solution scanned with blank electrodes and referenced with Ag/AgCl electrode.

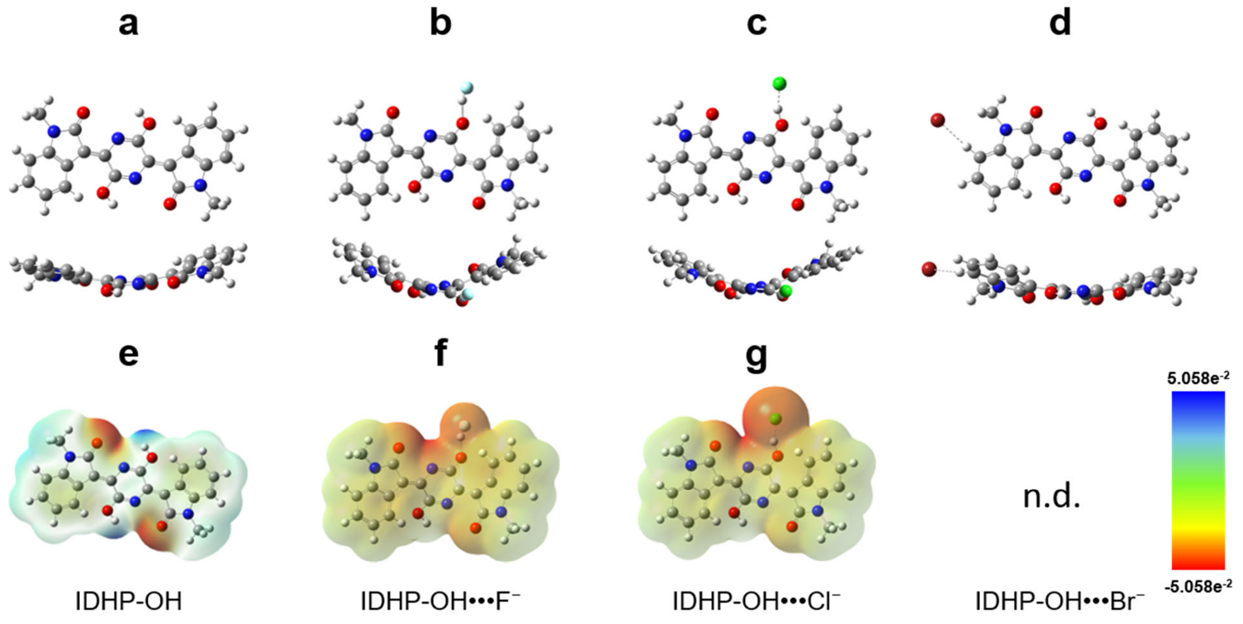


$d_{H\cdots F}$: 0.97 Å
EB: -452.37 kJ $mol^{-1}$

$d_{H\cdots Cl}$: 1.74 Å
EB: -116.49 kJ $mol^{-1}$

**Figure S16.** (a)-(d) DFT simulated molecular geometries of IDHP-OH and its complexes with fluoride, chloride, and bromide ions. (e)-(g) The ESP surfaces of energy-optimized IDHP-OH and its complexes with fluoride, chloride, and bromide ions together with the H-F and H-Cl bond lengths and binding energy (EB) values. Note: The geometry of IDHP-OH•••$Br^-$ could not be optimized due to the thermodynamically unfavorable interaction between the bromide ion and IDHP-OH, and therefore its ESP surface could not be obtained.

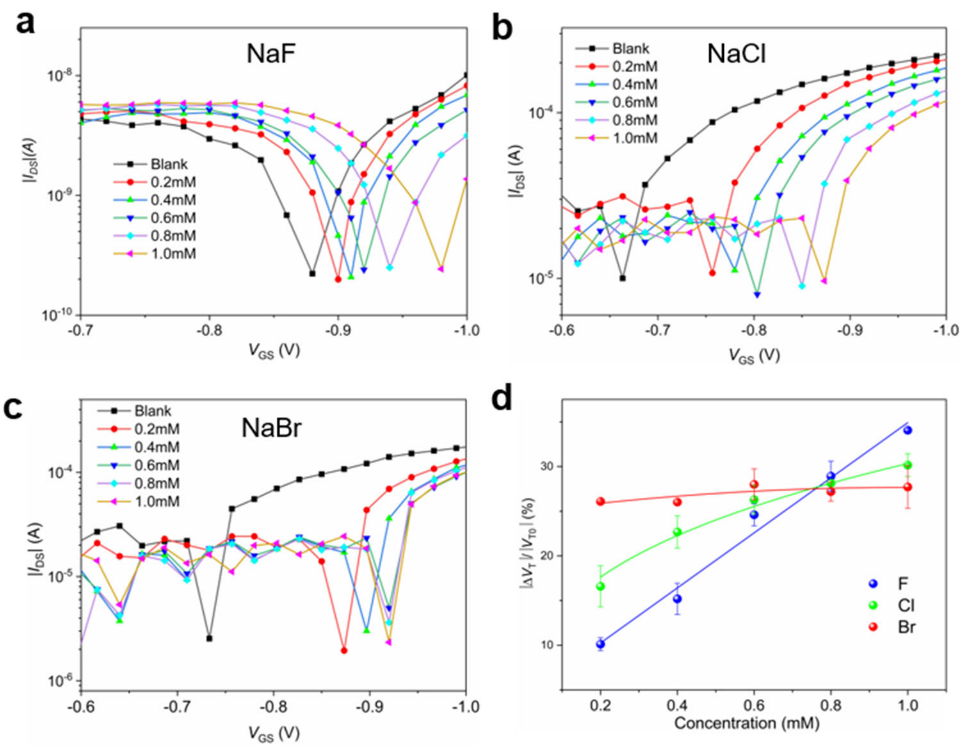


**Figure S17.** (a)-(c) Transfer characteristics of WG-OFET devices scanned at different sodium halide concentrations. (d) Changes in threshold voltages of WG-OFET devices at different sodium halide concentrations (average values from five devices per data point).

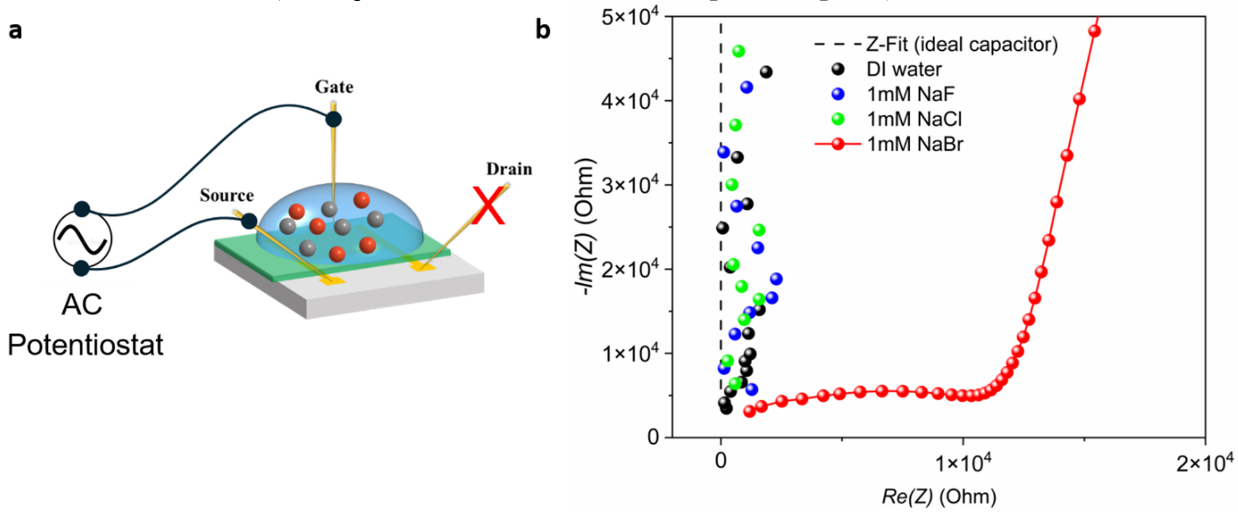


**Figure S18.** (a) EIS measurement setup and (b) Nyquist impedance profiles of PIDHPTT-based WG-OFET devices obtained measuring the impedance between the source and gate electrodes with DI water or 1 mM NaF, NaCl, or NaBr aqueous solution covering the interdigitated source/drain electrodes.

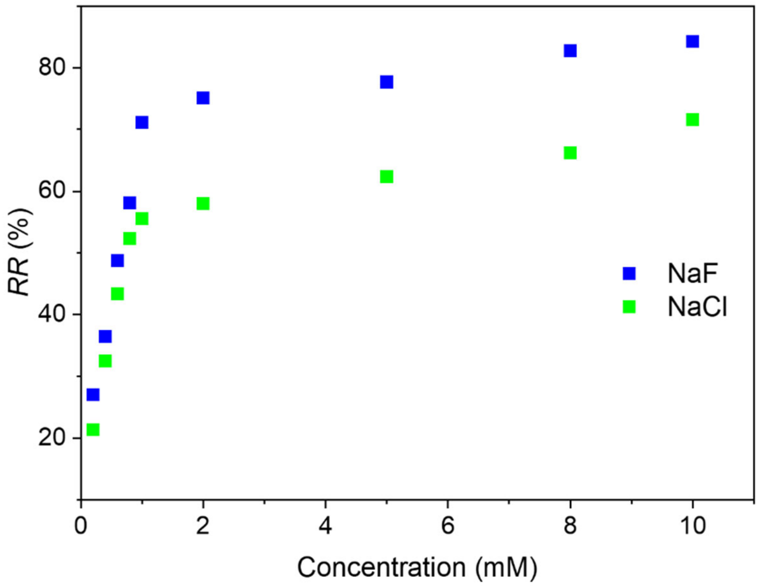


**Figure S19.** *RR* of PIDHP-based WG OFET sensors to fluoride and chloride ions in a concentration range of 0.2 mM to 10 mM.

## 5. Reference

[1] S. J. Jeon, N. Zhao, Y. Yuan, Y. Li, *Adv. Mater. Technol.* **2024**, 2301542.
[2] J. Pommerehne, H. Vestweber, W. Guss, R. F. Mahrt, H. Bässler, M. Porsch, J. Daub, *Adv. Mater.* **1995**, *7*, 551–554.
[3] L. Guo, H. Sato, T. Hashimoto, Y. Ozaki, *Macromolecules* **2010**, *43*, 3897–3902.
[4] M. B. Ibrahim, H. Y. Habib, R. M. Jabrah, *Rev. Compos. Matér. Avancés* **2020**, *30*, 133–141.
[5] J. Zhao, *Fibers Polym.* **2013**, *14*, 59–64.
[6] S. Attia, M. Schmidt, C. Schröder, P. Pessier, S. Schauermann, *Angew. Chem.* **2018**, *130*, 16901–16906.
[7] M. Xiang, M. Jiang, L. Feng, *Macromol. Rapid Commun.* **1995**, *16*, 477–481.
